# Bibliometric and visual analysis of neutrophil extracellular traps from 2004 to 2022

**DOI:** 10.3389/fimmu.2022.1025861

**Published:** 2022-10-21

**Authors:** Yantong Wan, Junyi Shen, Jiafu Ouyang, Peng Dong, Yinghao Hong, Lixin Liang, Jinghua Liu

**Affiliations:** ^1^ Guangdong Provincial Key Laboratory of Proteomics, Department of Pathophysiology, School of Basic Medical Sciences, Southern Medical University, Guangzhou, China; ^2^ The Second Clinical Medical College, Southern Medical University, Guangzhou, China; ^3^ College of Anesthesiology, Southern Medical University, Guangzhou, China

**Keywords:** Nets, neutrophil, VOSviewer, CiteSpace, visual analysis

## Abstract

**Background:**

Neutrophil extracellular traps (NETs) are specialized structures formed by neutrophils that were initially found to be important in killing pathogenic bacteria during infection. With the development of related research, the relationship between NETs and diseases such as sepsis, cancer, and systemic lupus erythematosus has received close attention. However, there is a lack of reports that comprehensively and objectively present the current status of NETs-related studies. Therefore, this study aims to visually analyze the current status and trends of NETs-related research by means of bibliometrics and knowledge mapping.

**Methods:**

NETs-related articles and reviews were retrieved using the Web of Science core collection subject search, and bibliometric analysis was performed in Excel 365, CiteSpace, VOSviewer, and Bibliometrix (R-Tool of R-Studio).

**Results:**

A total of 4866 publications from 2004 to 2022 were included in the bibliometric analysis. The number of publications shows an increasing trend from year to year. Collaborative network analysis shows that the United States and Germany are the most influential countries in this field, with the highest number of publications and citations. The journal with the most publications is Frontiers in Immunology. Brinkmann Volker is an authoritative author in this field, and his publication “Neutrophil extracellular traps kill bacteria” is the most frequently cited. The literature and keyword analysis shows that the relationship between NETs and diseases (hematological diseases, sepsis, cancer, etc.) and cell death (apoptosis, necroptosis, pyroptosis, etc.) is a popular research topic. Currently, NETs and SARS-CoV-2-related studies are at the forefront of the field.

**Conclusion:**

This study is the first to visualize the research in NETs-related fields using bibliometric methods, revealing the trends and frontiers of NETs research. This study will provide valuable references for scholars to find research focus questions and partners.

## Introduction

Neutrophils are the most abundant leukocytes in the human blood and play an essential role in the body’s resistance to infections caused by various pathogenic microorganisms (1). In addition to the clearance of pathogens by phagocytosis (2) and degranulation (3), neutrophil extracellular traps (NETs), first reported in 2004, are considered another mechanism of neutrophil antibacterial activity (4). NETs are reticular structures produced by neutrophils and are composed mainly of DNA and protein components (histones, granzymes and peptides) (4, 5). The generation of NETs is usually accompanied by a specific cell death form named NETosis (6). In the presence of NETs stimulants such as phorbol myristate acetate (PMA), protein kinase C is first activated, which promotes activation of reduced nicotinamide adenine dinucleotide phosphate (NADPH) oxidase complexes which in turn produces reactive oxygen species (ROS) (7, 8). Hydrogen peroxide, a type of ROS, is believed to mediate the dissociation of “azurosomes”, protein complexes formed in the membranes of azurophil granules and involving 8 different types of enzymes (9). Subsequently, the serine proteases (neutrophil elastase -NE, cathepsin G and azurocidin) and myeloperoxidase (MPO) in azurosomes are released into the cytosole and migrated to the nucleus where, together with Peptidyl-arginine deiminase 4 (PAD4), they promote citrullination of histones, ultimately leading to chromatin decondensation (10–12). Under the promotion of ROS, the nuclear membrane is gradually damaged and separated, and chromatin is released extracellularly through the membrane pores (13). Eventually, the cell membrane is cleaved, and NETs-related substances are released from the cytoplasm to the extracellular compartment (8). This type of NETs formation is called suicidal NETosis (14). Notably, NETs release may also proceed without neutrophil’s cell death (14). The formed NETs can trap and immobilize invading pathogens, including bacteria, viruses, and fungi (4). The NETs component contains antimicrobial proteins such as calprotectin, thereby leading to the killing of the trapped pathogens (15). Therefore, NETs formation is a new innate immune response (4). In addition, although NETs are beneficial for pathogen clearance, they have adverse effects on the organism (16, 17). Much evidence suggests that excess NETs are strongly associated with the development and progression of various diseases, including hematologic disorders (18), sepsis (19), systemic lupus erythematosus (SLE) (20), and tumors (21).

Bibliometric analysis is a method that uses mathematical and statistical methods to review and analyze studies in a specific field of research over a specific period, both qualitatively and quantitatively (22). This method focuses on countries, institutions, journals, authors, and keywords related to research in a specific field, providing readers with an objective view of trends and frontiers in the field (23, 24). Bibliometric analysis has been used in many research areas, including innate immunity (25), pyroptosis (26), ferroptosis (27), and other areas closely related to NETs. Despite the rapid development of NETs-related research in the last two decades, there is still a lack of bibliometric analyses related to the field of NETs. Therefore, this study aims to analyze the overall situation of NETs-related research and identify the research trends and frontier hotspots in the past two decades by using two bibliometric software programs, VOSviewer and CiteSpace, which may provide a reference for researchers to understand the corresponding fields and find collaborations.

## Materials and methods

### Data sources

The data for the metrological analysis of this study were obtained from the Web of Science Core Collection (WOSCC), a comprehensive, standardized database widely used in academia (28). In WOSCC, TS stands for Topic Sentence. The search formula used in this study was set to “TS= (neutrophils OR neutrophil) AND TS= (“NETs” OR “neutrophil extracellular traps” OR “neutrophil extracellular trap” OR “netosis”). The search period was limited to January 1, 2004 to July 10, 2022. Only “Article” and “Review” were selected as article types, and the language was limited to English, resulting in 4866 articles. The results were exported as plain text files in txt and CSV formats, according to the above formula for searching on WOSCC. The search was completed on July 10, 2022, to prevent data bias due to database updates.

### Data analysis and visualization

CiteSpace, developed by Chaomei Chen, is currently the most widely used software for bibliometric analysis (29). We used CiteSpace 6.1. R2 Advanced visualization to analyze country distribution and collaboration, the dual-map overlay of journals, institutional distribution, subject area distribution, keyword timeline graphs, reference collaboration and literature bursts. VOSviewer was developed by Nees Jan van Eck et al. and is mainly used for bibliometric network graph analysis (30). We used VOSviewer 1.6.18 to visually analyze country distribution, institution distribution, author distribution and collaboration and keyword collaboration. The clustering, wich relies on the similarity matrix and VOS mapping technique, was completed automatically and the corresponding labels were then added by the authors according to the content. In addition, we used Bibliometrix (R-Tool of R-Studio) (31) to visually analyze the country distribution, references and keywords, and Microsoft Excel 365 to show the publication and citation trends of the literature over the years. Finally, we used MATLAB [R2018a (9.4.0.813654)] software to predict the number of NETs-related publications. All raw data used in this study were obtained from public databases and therefore did not require ethical review.

## Results

### Annual publications and citation trends

**Figure 1:**
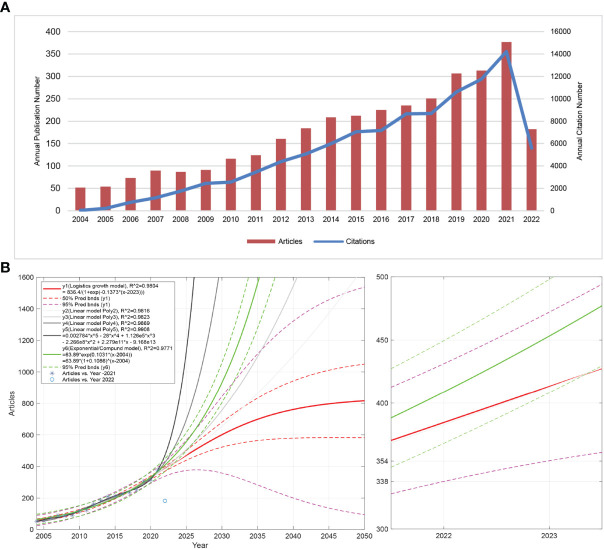
Description of NETs-related publication volume and citation frequency and forecast of publication volume. **(A)** The number of NETs-related publications and citation frequency for each year from 2004 to 2022, with the overall increasing trend of NETs-related publications and citation frequency reaching the maximum in 2021 (2022 data only until July 10). The number of publications fluctuates between 50-377, with a maximum value in 2021 and an average of more than 240 in the last decade (2012 to 2021). An overall upward trend in citation frequency is observed. **(B)** The left panel shows the forecast of NETs-related publications from 2022 to 2050, and the right panel shows the forecast of publications in 2022. y1 is the logistic growth model, y2, y3, y4, y5 is the linear model, and y6 is the general model. R^2 is the coefficient of the model. The larger the R^2 (close to 1), the better the fitted regression equation is. “Pred bnds” represents the predicted upper and lower bounds.

**Figure 1A** shows the annual publication volume and annual citation frequency of related articles from 2004 to 2022. In general, the number of NETs-related annual publications shows an increasing trend, with a decrease in 2008 and an increase in all other years. The year with the highest number of publications is 2021, with 377 articles. Overall, the annual citation frequency of NETs-related literature showed an increasing trend, with a more moderate increase in 2009-2010, 2015-2016, and 2017-2018. The 2021 literature had the highest annual citation frequency of 14,213, with the highest increase of 20.78% for all years. **Figure 1B** shows the logistic, linear, and general prediction model curves of article volume fitted by MATLAB software. The left panel shows the fitted curves for NETs-related article volume from 2004 to 2022 and the predicted curves for article volume from 2022 to 2050. The red curve y1 has a good fit (R2 = 0.9804), and the purple dashed line is the 95% prediction bounds for y1. The curves y2, y3, y4, and y5 all have R^2 values above 0.98, indicating a good fit. The results show that the expected peak annual publication volume of NETs-related research in the future may be approximately 800, and the peak time may be after 2050. The right panel shows the predicted curve of NETs-related publication volume in 2022. The results show that NETs-related research articles may exceed 425 in 2022.

### Distributions of countries/regions

**Figure 2:**
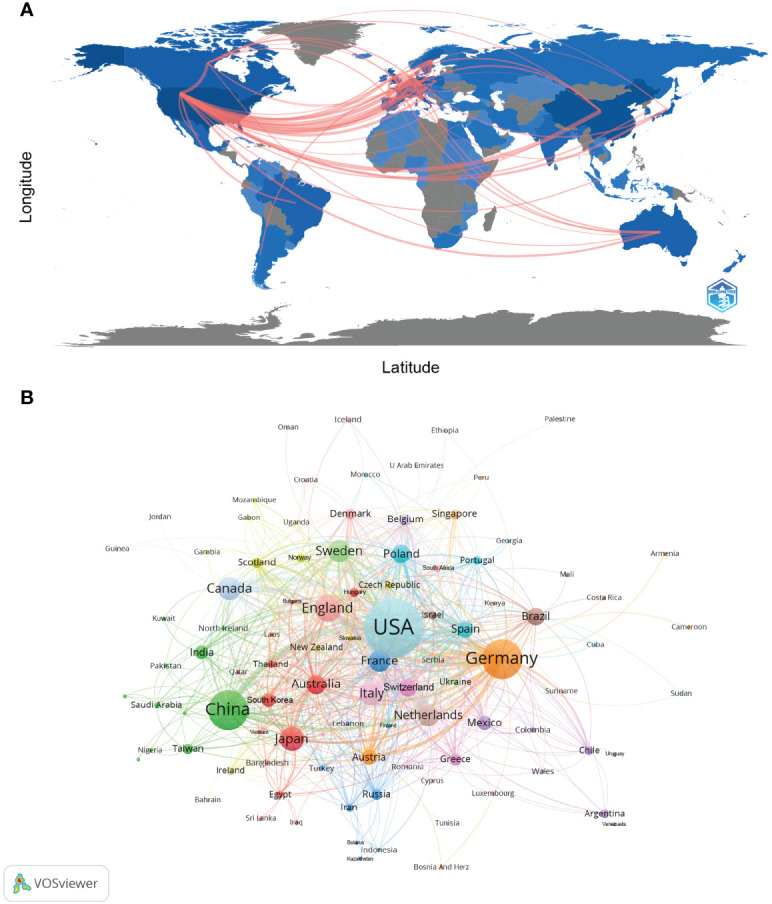
Analysis of NETs-related country/region. **(A)** Countries/regions involved in NETs-related research. The links between countries/regions indicate their collaborations and connections. **(B)** Analysis of collaborative network visualization of countries/regions in VOSviewer. The figure shows the countries/regions with more than 1 number of documents. The nodes of different colors represent the countries/regions with different clusters, and the size of the nodes indicates their node sizes.

Currently, there are 99 countries/regions participating in the study of NETs, mainly concentrated in the Northern Hemisphere. It is worth noting that the links between countries/regions are mainly concentrated between North America and Europe, North America, and East Asia, with strong links between Oceania and North America and Europe (**Figure 2A**). **Table 1** shows the top 10 countries/regions in terms of number of publications, the corresponding frequency of citations and centrality. The betweenness centrality of countries/regions measures the importance of the position of the countries/regions in the network. The USA published the most documents (1555), followed by China (738) and Germany (714). The USA has the highest citation frequency (93856), followed by Germany (49879). The citation frequency of all other countries/regions is less than 20,000.

**Table 1:** Top 10 countries/regions in terms of number of publications, the corresponding frequency of citations and centrality.

Rank	Countries/regions	publications	Citations	Centrality
1	USA	1555	93856	0.24
2	China	738	15214	0.05
3	Germany	714	49879	0.26
4	England	342	19846	0.18
5	Italy	280	12031	0.10
6	Japan	271	8594	0.04
7	Canada	249	19170	0.16
8	Sweden	224	12781	0.12
9	Netherlands	220	10669	0.10
10	France	216	12552	0.08

**Figures 2B** and **Supplementary Figure 1** show the international cooperation of the top 20 countries by publication volume. The results of the global collaboration network analysis show that countries and regions are roughly divided into 10 clusters in VOSviewer according to the closeness of collaboration, which are represented by different colors (**Figure 2B**). Each node in CiteSpace represents a country/region, and the radius of the node increases with its contribution to NETs research (**Supplementary Figure 1**). The connections between nodes indicate the collaborative relationship between individual countries and regions, and the thickness of the links is positively correlated with the depth of collaboration. The betweenness centrality of a node indicates its strength of association with other nodes, which is proportional to the size of the surrounding purple ring. The larger the purple circle, the larger the value of betweenness centrality. The USA and Germany are the main research centers of NETs and have close cooperation with several countries, such as England, Canada, China, and Australia.

### Distribution by institutions

**Table 2** and **Supplementary Table 1** shows the top 10 institutions in terms of number of publications, frequency of citations and the corresponding centrality. The institution with the highest number of publications is Harvard Medical School (127), followed by the University of Michigan (119). The top ten institutions in terms of number of publications are from the United States with five, followed by Sweden with two. The most frequently cited institution is the Max Planck Institute for Molecular Biomedicine (17982), followed by Harvard University (12087) and the University of Michigan (10752). The top 10 most cited institutions are from the United States with 7, followed by Germany with 2. Notably, The University of Amsterdam (0.33), The Beatson Institute for Cancer Research (0.31), The Baylor College of Medicine (0.29), The University Medical Center Mainz (0.27), and several other institutions show high centrality, which implies that these institutions occupy a significant position in research in the field of NETs.

**Table 2:** Top 10 institutions in terms of number of articles issued and the corresponding centrality.

Rank	Institution	Publication	Centrality
1	Harvard Medical School	127	0.02
2	University of Michigan	119	0.07
3	Karolinska Institute	83	0.13
4	Lund University	73	0.03
5	Harvard University	68	0.10
6	University of Toronto	67	0.09
7	University of Amsterdam	64	0.33
8	University of California, San Diego	60	0.12
9	Shanghai Jiao Tong University	57	0.02
10	Boston Children’s Hospital	52	0.05

**Figure 3:**
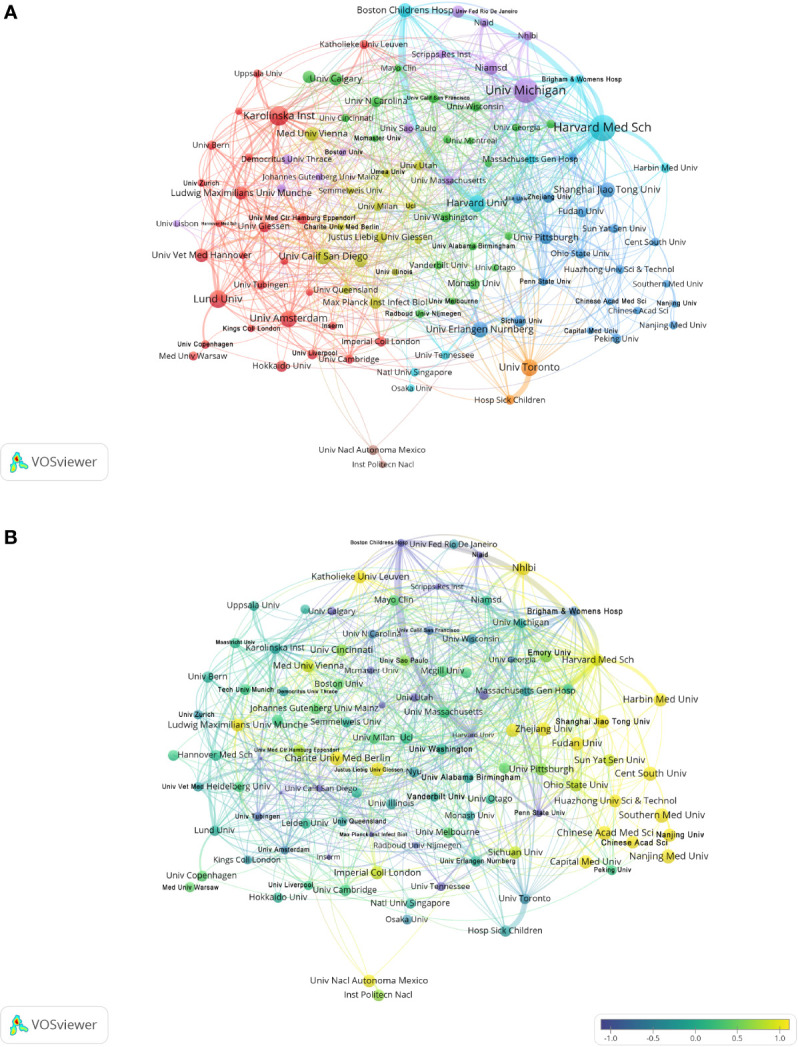
Analysis of NETs-related institution. **(A)** Analysis of collaborative network visualization of institutions in VOSviewer. The figure shows the institutions with more than 5 documents. The nodes of different colors represent the institutions of different clusters, and the size of the nodes indicates the frequency of their occurrence. **(B)** Analysis of the number of articles published by institutions in recent years. The recent 5 years heat value of each institution is obtained by dividing the number of publications in recent 5 year by the total number of publications.

The analysis by research institutions aims to understand the global distribution of NETs-related research and provide opportunities for cooperation. In VOSviewer, institutional cooperation is divided into 8 closely related blocks (**Figure 3A**). **Figure 3B** shows the ratio of institutional publications to total publications in the past five years, generated by dividing the number of NET-related publications in each institution over the past five years by their total number of publications from 2004 to 2022. **Figure 3B** shows the ratio of institutional publications to total publications in the past five years. The color bias towards yellow means a higher ratio, indicating that these institutions are emerging forces in the field of NETs; the color bias towards purple means a lower ratio, indicating that these institutions have relatively a little research in the field of NETs in recent years. The results show that the number of studies conducted by Harvard Med Sch, Shanghai Jiao Tong University, Harbin Medical University and other institutions has increased significantly in the past five years. In contrast, Boston Children’s Hospital, University of California San Diego, Max Plank Institute for Infection Biology and other institutions have conducted relatively few studies in the past five years. In CiteSpace, the University of Michigan is the most productive institution in the institutional cooperation network, but its centrality is low. In contrast, institutions such as the University of Amsterdam, University of California San Diego, and Karolinska Institution have higher centrality, indicating that they have extensive collaborations with academic institutions around the world (**Supplementary Figure 2**).

### Distribution of authors

Co-cited authorship analysis refers to the literature of two authors being cited by a third author simultaneously. A higher co-citation frequency indicates closer academic interest and research density (32). The analysis of the authors with the highest number of publications and co-citation frequencies in NETs-related research can visually reflect the research strength of the authors and NETs-related research hotspots. **Table 3** and **Supplementary Table 2** shows the top 10 authors in the number of publications, frequency of co-citations, the corresponding institutions and the corresponding total link strength. The author with the highest number of publications is Kaplan Mariana J. (National Institutes of Health, USA) (67), followed by Herrmann Martin (University Hospital Erlangen, Germany) (51), Knight Jason S. (University of Michigan, USA) (49) and Maren von Köckritz-Blickwede (University of Veterinary Medicine,Germany) (47). The most frequently co-cited author is Brinkmann Volker (Max Planck Institute, Germany) (4008), followed by Tobias A. Fuchs (New York University, USA) (2621) and Venizelos Papayannopoulos (The Francis Crick Institute, UK) (1690). It is worth noting that Brinkmann Volker has a high influence in this field in terms of both citations and co-citations.

**Table 3:** Top 10 authors in terms of number of publications, the corresponding institutions and total link strength.

Rank	Author	Publications	Institutions	Total link Strength
1	Kaplan, Mariana J.	67	National Institutes of Health (USA)	247
2	Herrmann, Martin	51	University Hospital Erlangen (Germany)	361
3	Knight, Jason S.	49	University of Michigan (USA)	257
4	Von Koeckritz-Blickwede, Maren	47	University of Veterinary Medicine (Germany)	149
5	Nizet, Victor	41	University of Rhode Island College of Pharmacy (USA)	112
6	Wagner, Denisa D.	36	Boston Children’s Hospital (USA)	136
7	Hermosilla, Carlos	32	Justus Liebig University (Germany)	129
8	Taubert, Anja	32	Justus Liebig University (Germany)	134
9	Nakazawa, Daigo	31	Hokkaido University (Japan)	166
10	Ritis, Konstantinos	31	Democritus University of Thrace (Greece)	191

**Figure 4:**
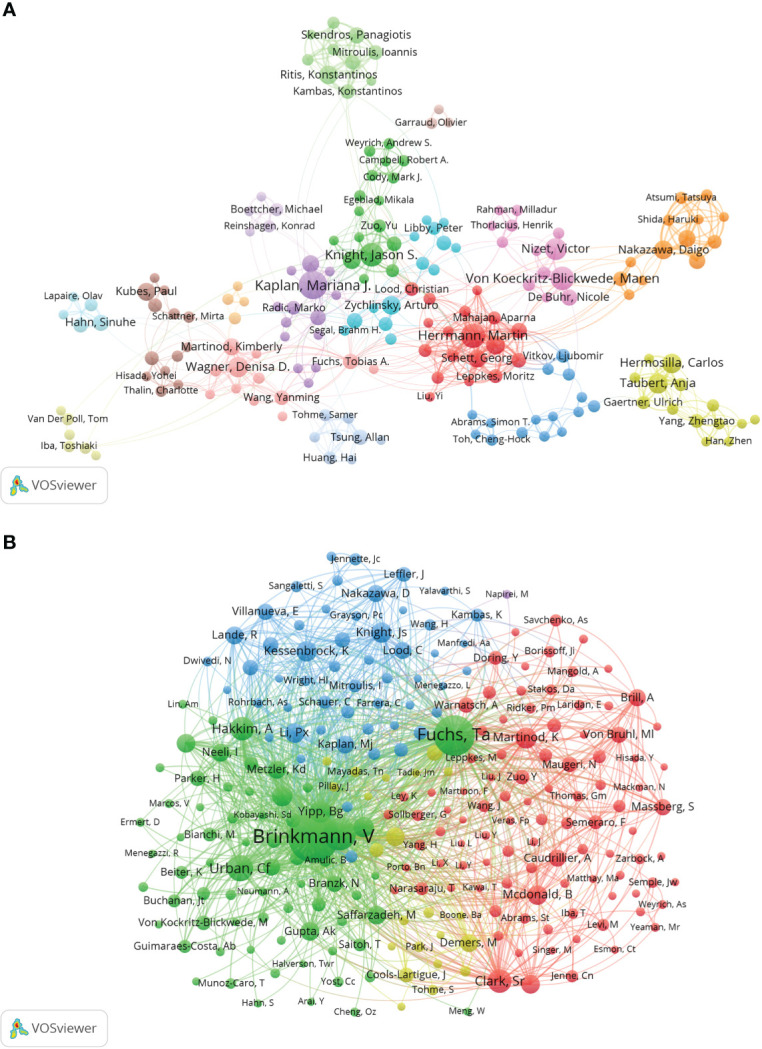
Analysis of NETs-related author. **(A)** Collaborative network visualization of authors in VOSviewer. The figure shows the authors with more than 8 documents. The nodes in different colors represent the authors in different clusters, and the size of the nodes indicates the frequency of their occurrence. **(B)** Analysis of collaborative network visualization of authors’ citations in VOSviewer. The size of the nodes indicates the frequency of their occurrence.

The collaborations of the authors of NETs-related literature are shown in VOSviewer (**Figure 4A**), which provides information for finding research partners and industry authorities. Herrmann Martin and Kaplan Marina J. are at the center of the collaborative network. Herrmann Martin is associated with Von Koeckritz-Blickwede Maren, Knight Jason S., Abrams Simon T. and Fuchs Tobia A. are actively collaborating, while Kaplan Marina J. is in close collaboration with Knight Jason S., Wagner Denisa D., Boettcher Michael and Fuchs Tobia A. The co-cited authorship network map shows that the research focus of the authors of NETs-related literature is highly homogeneous (**Figure 4B**). The authors are mainly divided into 4 clusters: Brinkmann V, Fuchs Ta, etc. (green); Martinod K, Clark Sr, Von Bruhl Ml, etc. (red); Knight Js, Kessenbrock K, Lande R, etc. (blue); Cools- Lartigue J, Demers M, et al. (yellow).

### Distribution of journals

We used the bibliometric online analysis platform to identify journals with high publication volume and impact in NETs-related fields. The journal’s impact factor (IF) and Journal Citation Reports (JCR) quartile reflect the journal’s influence. The journals with the top 25% (including 25%) of IF are in JCR quartile 1(Q1), and top 25%-50% (including 50%) of IF are in JCR quartile 2(Q2). **Table 4** and **Supplementary Table 3** shows the top 10 journals in the number of articles, frequency of co-citation, corresponding IF (JCR2021) and JCR quartile. The journal with the highest number of publications is Frontiers in Immunology (8.786, Q1) (388), followed by the International Journal of Molecular Sciences (6.208, Q1) (129), PLOS One (3.752, Q2) (109), and Scientific Reports (4.996, Q2) (109). Among the top ten journals in terms of the number of publications, five journals are distributed in the Q1 JCR, and eight have the IF above 5. The most frequently co-cited journals are Blood (25.476, Q1) (14576) and Journal of Immunology (5.446, Q2) (13208). Among the top 10 journals in co-citation frequency, eight journals are distributed in Q1 JCR and six journals have an IF over 10. It is worth noting that 4 of the top 10 journals in terms of publication volume are also among the top 10 journals in terms of co-citation frequency, including Frontiers in Immunology, Plos One, Journal of Immunology and Blood, indicating a strong influence of these journals.

**Table 4:** Top 10 journals in terms of number of publications, corresponding IF (JCR 2021) and JCR quartile.

Rank	Journal	Publications	IF (JCR2021)	JCR quartile
1	Frontiers In Immunology	388	8.786	Q1
2	International Journal of Molecular Sciences	129	6.208	Q1
3	Plos One	109	3.752	Q2
4	Scientific Reports	109	4.996	Q2
5	Journal of Immunology	76	5.446	Q2
6	Journal of Leukocyte Biology	66	6.011	Q2
7	Cells	63	7.666	Q2
8	Blood	62	25.476	Q1
9	Thrombosis And Haemostasis	48	6.681	Q1
10	Journal of Thrombosis And Haemostasis	45	16.036	Q1

**Figure 5:**
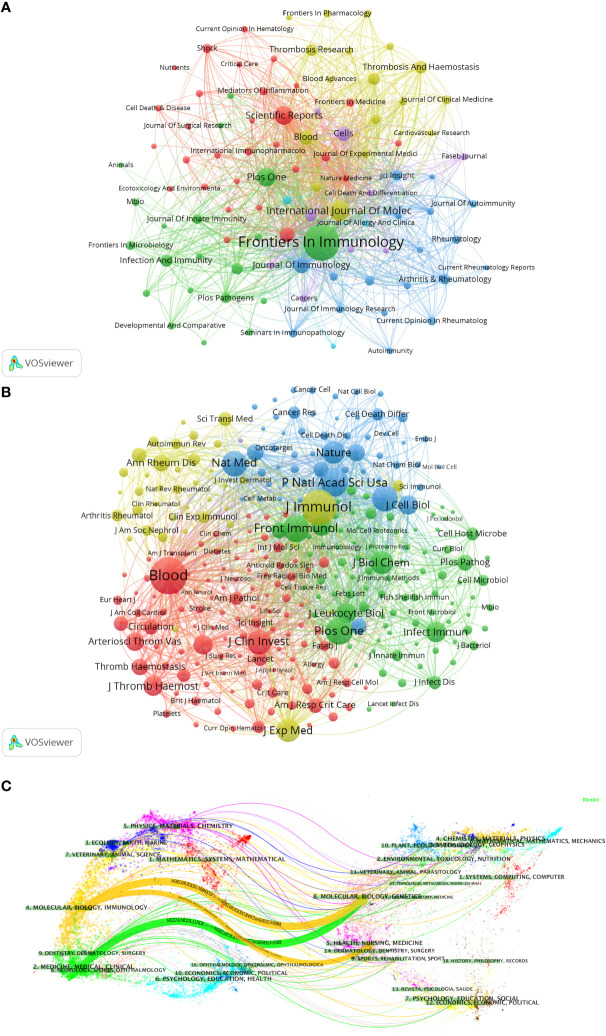
Analysis of NETs-related journal. **(A)** Analysis of collaborative network visualization of journals in VOSviewer. The figure shows the journals with more than 10 documents. The nodes in different colors represent the journals in different clusters, and the size of the nodes indicates the frequency of their occurrence. **(B)** Analysis of collaborative network visualization of journals’ citations in VOSviewer. The size of the nodes indicates the frequency of their occurrence. **(C)** The dual-map overlay of journals. Citing journals are on the left, cited journals are on the right, and colored paths indicate citation relationships.

The visualization in VOSviewer shows the journals in which NETs-related literature was published and the relationships between them (**Figure 5A**). The clustering is based on the similarity of the journals and is divided into 5 categories overall: the blue cluster has studies focused on autoimmunity (Journal of Autoimmunity, Rheumatology, etc.); the green cluster has studies focused on immunity (Frontiers in Immunology, Infection And Immunity, etc.); yellow clusters are focused on clinical research and treatment as well as blood-related fields (Journal of Clinical Medicine, Thrombosis Research, etc.); red clusters are focused on critical care medicine (Shock, Critical Care, Journal of Surgical Research, etc.); and the studies in the purple cluster are mainly in the field of cell biology (Cells, etc.). Based on the co-cited frequency, these journals are classified into 4 clusters that tend to have similar research directions (**Figure 5B**). The red cluster is focused on hematology-related areas (Blood, Journal of Thrombosis and Hemostasis, etc.); the green cluster is focused on immunity (Frontiers in Immunology, Journal of Innate Immunity, etc.); the blue cluster is mainly in the field of biochemistry and molecular biology (Journal of Cell Biology, Cell Death and Differentiation, etc.); and the yellow cluster is mainly in the field of autoimmunity (Annals of The Rheumatic Diseases, Autoimmunity Reviews, etc.).

We used knowledge flow analysis to explore the evolution of knowledge citations and co-citation between citing and cited journals (33). The dual-map overlay of journals shows the topic distribution, changes in citation trajectories, and shifts in research centers across academic journals (**Figure 5C**) **(** 33, 34). The labels on the left of the dual map represent citing journals, and the labels on the right represent cited journals. A colored curve of citation connections originating from the citing map and pointing to the cited map shows the context of the citation (33). Citing journals are mainly from MOLECULAR, BIOLOGY, IMMUNOLOGY, MEDICINE, MEDICAL, and CLINICAL, called research frontiers. The cited journals are mainly from MOLECULAR, BIOLOGY, GENETICS, HEALTH, NURSING, MEDICINE, DERMATOLOGY, DENTISTRY, and SURGERY, called the knowledge base.

### Keyword analysis

**Figure 6:**
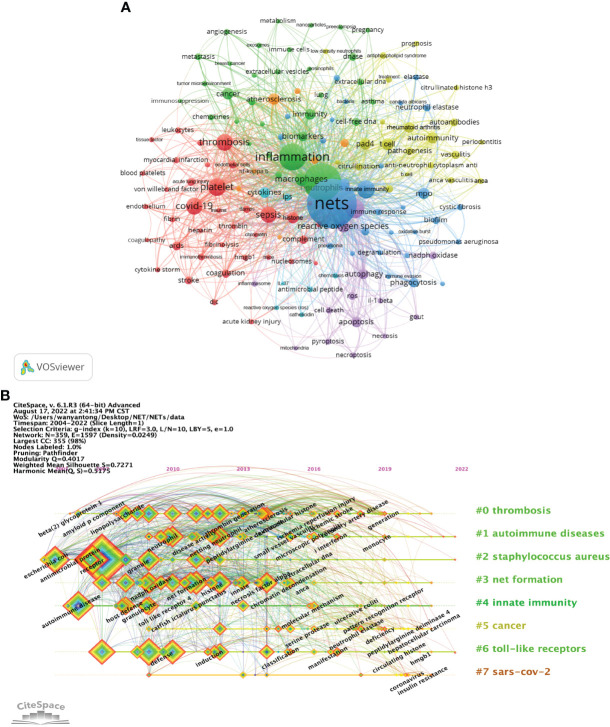
Analysis of NETs-related keyword. **(A)** Collaborative network visualization of keywords in VOSviewer. The figure shows the keywords with more than 15 occurrences. The nodes of different colors represent the keywords of different clusters, and the size of nodes indicates their frequency. **(B)** Timeline view of keywords. Each horizontal line represents a cluster. The smaller the number is, the larger the cluster, with #0 being the largest cluster. Nodes size reflects co-citation frequency, and the links between nodes indicate co-citation relationships. Nodes occurrence year is the time when they were first co-cited.

As an overview of the core content of the article, keywords can be used to analyze the frontiers of NETs research. **Table 5** shows the top 20 keywords by frequency. The most frequently occurring keyword is “nets” (1711), followed by “neutrophils” (1002). In addition, “inflammation” (1314) and “netosis” (691) are frequent keywords, indicating that their corresponding fields are popular in NETs-related research. A co-occurrence network diagram of keywords is visualized in VOSviewer (**Figure 6A**). The connecting lines between different keywords indicate that they have co-occurrence relationships. The keywords are clustered according to the research direction and roughly divided into 5 categories: keywords in the blue cluster are related to physiological or pathological phenomena (phagocytosis, degranulation, etc.) and are intracellular and extracellular substance related (MPO, biofilm, etc.). The keywords in the green cluster are related to inflammation (inflammation, macrophages, immune cells, etc.). Keywords in the red cluster are related to cardiovascular (thrombosis, platelet, etc.) and critical medicine (COVID-19, sepsis, etc.). The keywords in the purple cluster are related to cell death (cell death, apoptosis, etc.). The keywords in the yellow cluster are related to autoimmunity (autoimmunity, autoantibodies, etc.). The light blue cluster (cytokines, lipopolysaccharide, etc.), and the orange cluster (atherosclerosis, etc.) are linked to several clusters, indicating that they are cross-cutting areas in each research direction.

**Table 5:** Top 20 keywords in terms of frequency of occurrence and the corresponding total link strength.

Rank	Keyword	Occurrences	Total link strength
1	nets	1711	3369
2	neutrophils	1002	2374
3	inflammation	517	1314
4	netosis	266	692
5	platelet	234	616
6	innate immunity	224	537
7	covid-19	200	460
8	thrombosis	197	575
9	sepsis	183	493
10	macrophages	119	356
11	systemic lupus erythematosus	101	243
12	reactive oxygen species	99	248
13	autoimmunity	96	254
14	infection	96	278
15	histone	95	249
16	atherosclerosis	93	290
17	cancer	93	286
18	pad4	90	246
19	mpo	89	216
20	apoptosis	88	248

In CiteSpace, the timeline graph shows the most frequently occurring keywords for each cluster over time (**Figure 6B**). The earliest and largest cluster is #0 (thrombosis). In this field, the earliest keywords include beta-2 glycoprotein I (beta2GPI) and thrombin generation, while ischemia reperfusion injury, ischemic stroke and coronary artery disease are the latest research targets. Another large cluster that emerged earlier is #1 (autoimmune diseases). In this field, keywords such as microscopic polyangiitis, interferon and monocytes are the research frontiers. It is worth noting that 3 of the 7 clusters are still in progress, namely, #1 (autoimmune diseases), #2 (staphylococcus aureus) and #6 (toll-like receptors), indicating that relevant research in this field is ongoing. In addition, #7 (SARS-COV-2) is the latest cluster; the main keywords are coronavirus and insulin resistance, which is a frontier hotspot of NETs-related research. The details of **Figure 6B** are provided in the **Supplementary Table 4**.

**Figure 7:**
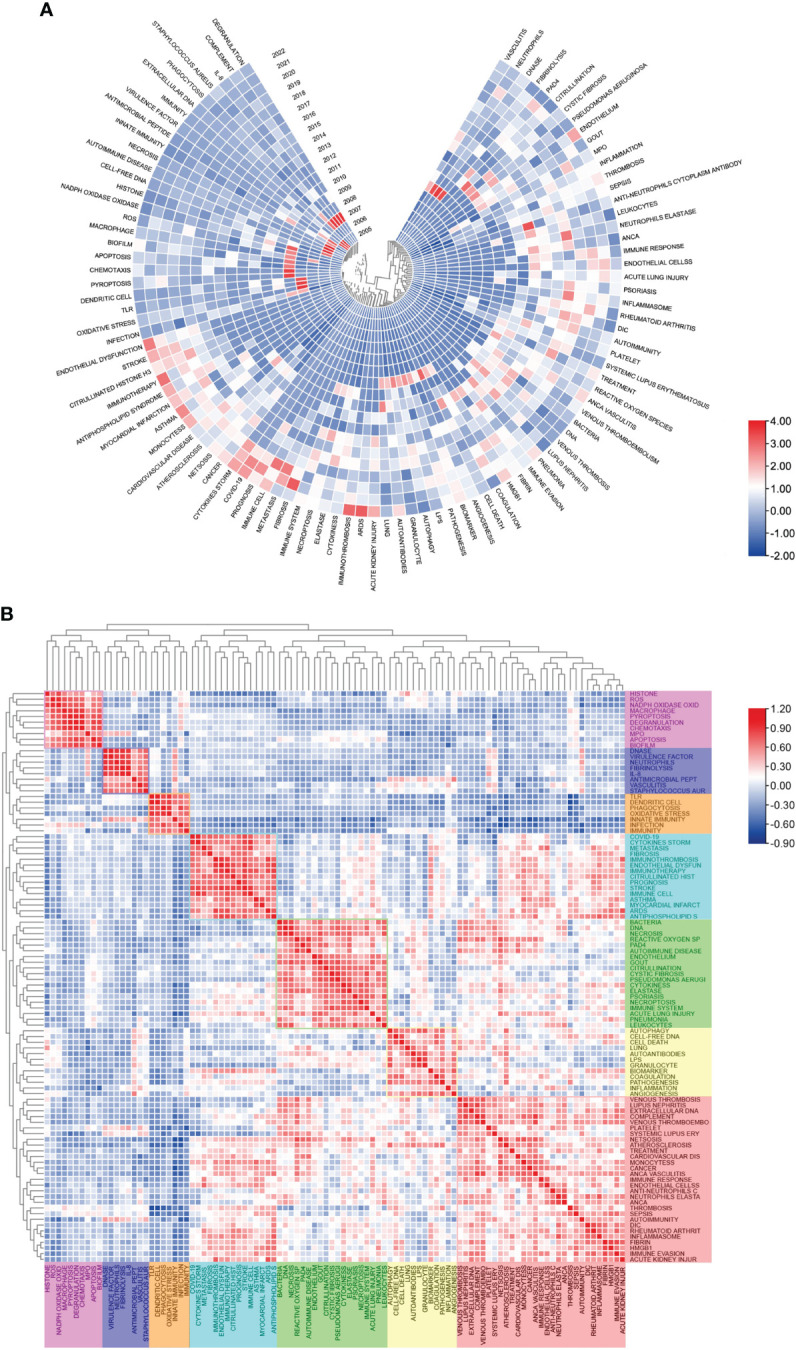
Heatmap analysis of NETs-related keywords. **(A)** Annual heatmap from 2004 to 2022. The annual heat value of each keyword is obtained by dividing the number of citations in that year by the total number of citations in that year. **(B)** Keyword relevance heatmap. Keywords with high popularity in similar time periods are clustered into one category and marked with different colors.

**Figure 7A** shows the keywords’ annual popularity (number of citations in the year/total citations in the year) from 2005 to 2022. Keywords such as phagocytosis and antimicrobial peptide have had relatively low annual popularity in recent years. In contrast, the annual popularity of keywords such as stroke, citrullinated histone, cytokines storm, and COVID-19 have been relatively high in recent years, suggesting that these keywords represent emerging frontier areas. **Figure 7B** shows the popularity correlation of keywords, where keywords with high popularity in similar periods are clustered into different clusters marked with different colors. The results show that there are 7 clusters: the pink cluster (histone, ROS, NADPH oxidase oxide, etc.), purple cluster (DNase, virulence factor, neutrophils, etc.), orange cluster (TLR, dendritic cell, phagocytosis, etc.), blue cluster (COVID-19, cytokine storm, fibrosis, etc.), green cluster (bacteria, DNA, necrosis, etc.), yellow cluster [autophagy, cell-free DNA, cell death, etc.)], and red cluster (venous thrombosis, lupus nephritis, extracellular DNA, etc.). This indicates that keywords within the same cluster have higher popularity in the same period.

### Highly cited reference analysis

**Table 6** shows the top ten articles in terms of citation frequency and average annual citation frequency. The most frequently cited article is “Neutrophil extracellular traps kill bacteria” (Brinkmann V, et al., 2004) (5334), which focuses on the composition of NETs and their bacteriostatic effect in experimental dysentery and spontaneous human appendicitis (4). Notably, this was the first paper in the field, discovering NETs, which justifies its very high number of citations. Next, “the novel cell death program leads to neutrophil extracellular traps” (Fuchs, Tobias A., et al., 2007) (1925), which describes a process of NETs formation: the nucleus is deformed, and chromatin is homogenized after stimulation of neutrophil NET components is mixed, followed by NADPH oxidase and ROS-mediated cell death accompanied by cell membrane rupture, which releases NETs to exert antimicrobial effects (8). The most frequently cited article on an annual basis is “Molecular mechanisms of cell death: recommendations of the Nomenclature Committee on Cell Death 2018” (Galluzzi Lorenzo, et al., 2018) (354.2). This article suggests defining Netotic cell death as ROS -dependent, regulated cell death confined to hematopoietic-derived cells and closely associated with NETs release (35). The second most frequently cited annual average is “Neutrophil extracellular traps kill bacteria” (280.7).

**Table 6:** Top 10 highly cited references.

Rank	Article Title	Source Title	Authors	Year	Cited	DOI
1	Neutrophil extracellular traps kill bacteria	SCIENCE	Brinkmann, V,et al.	2004	5334	10.1126/science.1092385
2	Novel cell death program leads to neutrophil extracellular traps	JOURNAL OF CELL BIOLOGY	Fuchs, Tobias A.,et al.	2007	1925	10.1083/jcb.200606027
3	Molecular mechanisms of cell death: recommendations of the Nomenclature Committee on Cell Death 2018	CELL DEATH AND DIFFERENTIATION	Galluzzi, Lorenzo,et al.	2018	1771	10.1038/s41418-017-0012-4
4	Molecular definitions of cell death subroutines: recommendations of the Nomenclature Committee on Cell Death 2012	CELL DEATH AND DIFFERENTIATION	Galluzzi, Lorenzo,et al.	2012	1713	10.1038/cdd.2011.96
5	Platelet TLR4 activates neutrophil extracellular traps to ensnare bacteria in septic blood	NATURE MEDICINE	Clark, Stephen R.,et al.	2007	1411	10.1038/nm1565
6	Extracellular DNA traps promote thrombosis	PROCEEDINGS OF THE NATIONAL ACADEMY OF SCIENCES OF THE UNITED STATES OF AMERICA	Fuchs, Tobias A.,et al.	2010	1349	10.1073/pnas.1005743107
7	The acute respiratory distress syndrome	JOURNAL OF CLINICAL INVESTIGATION	Matthay, Michael A.,et al.	2012	1127	10.1172/JCI60331
8	Neutrophil elastase and myeloperoxidase regulate the formation of neutrophil extracellular traps	JOURNAL OF CELL BIOLOGY	Papayannopoulos, Venizelos,et al.	2010	1069	10.1083/jcb.201006052
9	Monocytes, neutrophils, and platelets cooperate to initiate and propagate venous thrombosis in mice *in vivo*	JOURNAL OF EXPERIMENTAL MEDICINE	von Bruehl, Marie-Luise,et al.	2012	1047	10.1084/jem.20112322
10	Netting neutrophils in autoimmune small-vessel vasculitis	NATURE MEDICINE	Kessenbrock, Kai,et al.	2009	1027	10.1038/nm.1959

**Figure 8:**
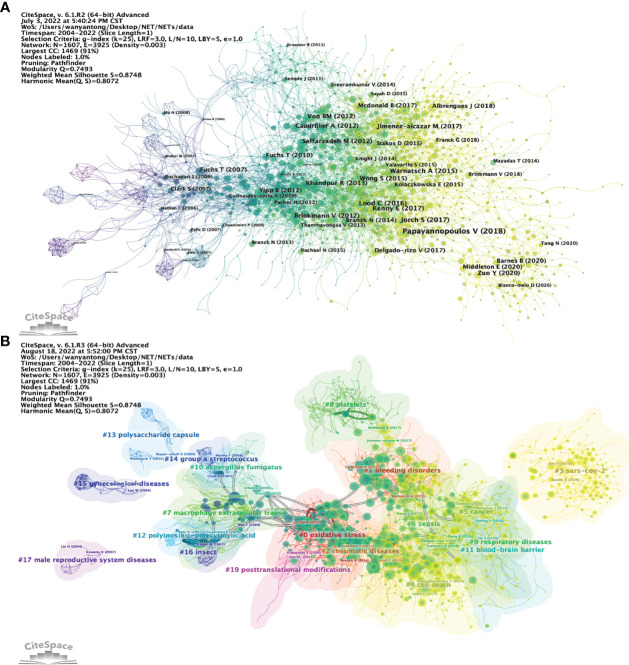
Analysis of NETs-related reference. **(A)** Analysis of the network of references in CiteSpace. Node size is proportional to the number of times the article is co-cited. **(B)** Clustering of references based on similarity between references, including #0 oxidative stress, #1 bleeding disorders, #2 rheumatic diseases, #4 cell death, #5 cancer, #9 respiratory diseases, #12 polyinosinic-polycytidic acid, #15 gynecological diseases, #16 insects, and #17 male reproductive system disease.

Article co-citation analysis analyzes the relationship between articles by analyzing the co-citation frequency of the articles (36). The relationship between studies is presented in CiteSpace, and the authors and years of the bursting articles with increased citation frequency are indicated in the figure (**Figures 8A, B** **)**. The clustering is based on the degree of association between the literature and was divided into 19 categories, which are indicated by different colors. The category with the highest number of published articles is #0, and the most common keyword in these articles is oxidative stress. In terms of timeline, the earliest research areas in the NETs field are three separate research clusters: #13 (polysaccharide capsule), #15 (gynecological diseases), and #17 (male reproductive system diseases), which together are developed into #14 (Group a Streptococcus), #7 (macrophage extracellular traps), and #19 (posttranslational modifications). Furthermore, #16 (insect) and #12 (polyinosinic-polycytidylic acid) are later independent research clusters; #16 developed mainly into clusters #10, #19 and #12 developed into clusters #7 and #0. After 2009, clusters #0 (oxidative stress) and #2 (rheumatic diseases) are closely related and then developed into three relatively discrete clusters, #1 (bleeding disorders), #6 (sepsis), and #4 (cell death). Subsequently, the closeness of the linkages between the studied areas further declined, and several relatively independent clusters emerged, including #5 (cancer), #11 (blood−brain barrier), #9 (respiratory diseases), and #3 (SARS-CoV-2). Notably, cluster #8 (platelets) originated separately from the study of Aslam R et al. (2006) in cluster #19 and eventually developed into a more independent line of research in areas such as cluster #1 (bleeding disorders).

**Figure 9:**
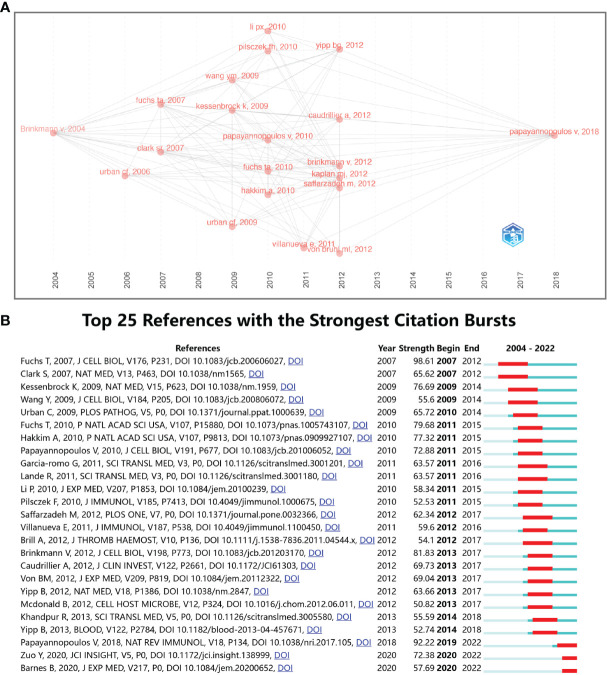
**(A)** Association between the top 20 citation bursts. **(B)** The top 25 references with the strongest citation bursts.

**Figure 9A** shows the relationship of the top twenty articles by citation frequency. The results show that “Neutrophil extracellular traps kill bacteria” published by Brinkmann V et al. in 2004 (4) received the most citations from other articles. Subsequently, 2010 (5 articles) and 2013 (6 articles) had the most highly cited articles, and these articles served as a link between the previous phase and the next phase. Ultimately, most of the articles are cited in “Neutrophil extracellular traps in immunity and disease” by Papayannopoulos V et al., 2018 (37). **Figure 9B** shows the top 25 references with the strongest citation bursts. The first two citation bursts occurred in 2007. They are titled “Novel cell death program leads to neutrophil extracellular traps” (8) and “Platelet TLR4 activates neutrophil extracellular traps to ensnare bacteria in septic blood” (38). It is worth noting that “Novel cell death program leads to neutrophil extracellular traps” is the paper with the strongest burst (Strength = 98.61), published by Tobias A Fuchs et al. in the Journal of Cell Biology in 2007, and its burst duration lasts until 2012. “Neutrophil extracellular traps in immunity and disease” by Venizelos Papayannopoulos, published in Nature Reviews Immunology, also had a high burst (Strength = 92.22). The results show that 2011 had the highest number of new citation bursts (7 times), followed by 2013 (5 times), which indicates that the high-burst papers in these two years caused a related research boom. There are two citation bursts with research directions for COVID-19 until 2020 (39, 40), which shows that NETs-related research is ongoing.

### Subject area analysis

CiteSpace analysis shows the citation relationship of NETs-related literature by subject area (**Supplementary Figure 3**). The literature related to the discipline “IMMUNOLOGY” has the highest number of citations, followed by “BIOCHEMISTRY & MOLECULAR BIOLOGY”, “CELL BIOLOGY “BIOCHEMISTRY & MOLECULAR BIOLOGY”, “CELL BIOLOGY”, “MICROBIOLOGY” and “MICROBIOLOGY”. “BIOCHEMISTRY & APPLIED MICROBIOLOGY”, “RADIOLOGY, NUCLEAR MEDICINE & MEDICAL IMAGING”, and “NEUROSCIENCES”, “ENDOCRINOLOGY & METABOLISM”, “ONCOLOGY”, “ONCOLOGY”. “BIOCHEMISTRY & MOLECULAR BIOLOGY”, “CELL BIOLOGY”, “MICROBIOLOGY”, “RESPIRATORY SYSTEM” and “CRITICAL CARE MEDICINE” are marked by purple circles, indicating that these disciplines have a greater influence in this field. The influence of these disciplines in this field is indicated by the purple circles around these disciplines.

## Discussion

CiteSpace 6.1. R2 Advanced, VOSviewer 1.6.18, and R-bibliometrix were used to analyze the data of 4866 articles on neutrophil extracellular traps between 2004 and 2022 from the Web of Science and to evaluate the spatial and temporal distributions, author contributions, core articles, research hotspots and frontiers of the field based on these data.

### General distribution

The analysis in this study is based on 4866 NETs-related articles from 1117 institutions with 22,373 authors in the WOSCC database from January 1, 2004, to July 10, 2022. The rapid increase in the number of articles indicates that NETs is attracting increasing attention. Brinkmann Volker et al. first reported the term “Neutrophil extracellular traps, NETs” in 2004, which began NETs-related research. The number of studies related to NETs has steadily increased over the past decade or so, with approximately three times as many publications in 2021 as in 2011.

In the country/region analysis, the two most important indicators are the number of publications and betweenness centrality. High centrality (>=0.10) means that these countries/regions act as “bridges” in the global collaborative network. As shown in **Tables 1**, **2** and **Figures 2**, **3**, the USA and Germany are the central countries for research in the NETs field. The United States has the highest number of publications and citation frequency, while Germany has the third highest number of publications and the second highest citation frequency. Five of the top ten institutions in terms of number of publications are from the United States; seven of the top ten institutions in terms of citation frequency are from the United States and two are from Germany, with Max Planck Institute for Infection Biology from Germany having a much higher citation frequency than other institutions. Germany’s centrality of 0.26 is the highest among all countries, followed by the USA with a centrality of 0.24, indicating its dominant position in global NETs research collaboration. In addition, countries such as the UK, China, Canada, and Switzerland are widely involved in NETs research and collaboration.

As seen in **Table 3** and **Figure 4**, Brinkmann Volker’s co-citation frequency ranks first and far exceeds that of other scholars, which indicates his outstanding influence in NETs-related fields. Brinkmann Volker is from Max Planck Institute for Infection Biology, Germany. In 2004, Brinkmann Volker et al. published the article “Neutrophil Extracellular Trap Kills Bacteria” in Science, which described for the first time an extracellular scaffold structure with DNA that can trap and kill pathogens and named it “Neutrophil extracellular traps, NETs”; it has been cited 5,334 times and is the most frequently cited article in this field. It is worth mentioning that this work was carried out under the guidance of Arturo Zychlinsky, who is the corresponding author of this article. Kaplan Mariana, J. is the top author in terms of the number of articles published and ranked third in citation frequency. Notably, Galluzzi Lorenzo’s team’s “Molecular mechanisms of cell death: recommendations of the Nomenclature Committee on Cell Death 2018” and “Molecular definitions of cell death subroutines: recommendations of the Nomenclature Committee on Cell Death 2012” ranked third in citation frequency and first in average annual citation frequency. The citation frequency of “Molecular definitions of cell death subroutines: recommendations of the Nomenclature Committee on Cell Death 2012” ranked fourth and also has a significant influence in related fields.

As shown in **Table 4** and **Figure 5**, Frontiers in Immunology is not only the journal with the highest number of publications but also one of the top 5 journals in terms of co-citation frequency. Blood received the highest number of co-citations and the second highest citation frequency, mainly due to the high number of relevant and highly cited articles published in the journal. Notably, among the top 10 journals in terms of co-citation frequency, there are 3 journals related to IMMUNOLOGY (J Immunol, Front Immunol, J Exp Med), excluding MULTIDISCIPLINARY SCIENCES. Two journals are related to BIOCHEMISTRY & MOLECULAR BIOLOGY-related journals (NATURE MEDICINE, J Biol Chem), and J Clin Invest is associated with MEDICINE, RESEARCH & EXPERIMENTAL, which is consistent with the dual-map analysis in **Figure 5C**.

### Hotspots and frontiers

Keyword analysis helps to understand the field frontier and hot content of NETs. In existing studies, the main keywords include “nets”, “neutrophils”, “inflammation”, “netosis”, “platelet”, “innate immunity”, “COVID-19”, “thrombosis”, and “sepsis” (**Table 5**), which are mainly related to NET formation and release and NETs-related diseases, suggesting that these keywords are popular topics for NETs. The co-occurrence network diagram shows that in past research, high-frequency keywords showed several popular research directions, including HEMATOLOGY (thrombosis, platelet, myocardial infarction, etc.), IMMUNOLOGY (immunity, autoimmunity, immunosuppression, etc.), CELL DEATH (apoptosis, necroptosis, pyroptosis, etc.), and INFLAMMATION (sepsis, COVID-19, etc.). Correspondingly, the timeline diagram analysis shows that #0 (thrombosis), #1 (autoimmune diseases), #2 (staphylococcus aureus), and #3 (net formation) are all larger clusters, indicating that they have higher heat. **Figure 7** shows that in recent years, INFLAMMATION, HEMATOLOGY, IMMUNOLOGY, and CELL DEATH remain hot research fields, and the keywords in each field represent hot new research directions. The heat of the emerging THERAPY field continues to increase, mainly including keywords such as immunotherapy and prognosis. It is worth noting that, similar to the interdisciplinary content of IMMUNOLOGY and HEMATOLOGY, the popularity of IMMUNOTHROMBOSIS continues to increase, indicating that interdisciplinary research has strong potential for popularity.

#### Neutrophil extracellular traps and diseases

The link between NETs and disease has received increasing attention. As shown in **Figure 8A**, the earliest popular research on NETs-related diseases mainly focused on cardiovascular diseases (#1 bleeding disorder, #2 rheumatic diseases), among which the important role of NETs in the process of thrombosis attracted much attention (41). “Extracellular DNA traps promote thrombosis” published by Tobias A Fuchs et al. elaborated the role of NETs in thrombosis from the perspective of platelet adhesion, activation, and aggregation (18). NETs can also promote thrombus formation by promoting thrombin generation (42) and activating thrombosis-related molecules (43). In addition, NETs play an important role in atherogenesis (44). In recent years, the relationship between NETs and sepsis, cancer, respiratory diseases, and other diseases has attracted increasing attention (45–48). Although NETs can clear pathogens from the body, there is evidence that excessive NETs in sepsis can exacerbate tissue damage (37), which may be related to elevated neutrophil-derived circulating free DNA (used to assess sepsis-related organ function) obstacles (49). Notably, the promotion of disseminated intravascular coagulation and thrombosis by NETs is also one of the mechanisms of sepsis development (50, 51). The relationship of NETs to cancer was first elucidated by Sivan Berger-Achituv et al., who found that intratumoral NETs in Ewing sarcoma were associated with poorer prognosis (52). NETs were subsequently shown to promote tumor development in many cancers, such as pancreatic (53), breast (54), and bowel cancer (55). Evidence suggests that tumor cells promote the release of NETs from neutrophils through stimulation, such as secretion of IL-8/CXCL8 and CXCR1/CXCR2 agonists, which provides an explanation for the link between NETs and cancer (56–58). Research on the relationship between NETs and lung diseases is a new hot spot in recent years, among which acute lung injury (ALI) and acute respiratory distress syndrome (ADRS) are very popular (59, 60). NETs were shown to be increased in ARDS, and this was closely associated with diminished macrophage phagocytosis (59). One possible explanation is that the increased NETs in ALI/ARDS affects the progression of ARDS by activating the pyroptosis of lung macrophages through inflammatory pathways (61). The relationship between NETs and cystic fibrosis (CF) lung disease is also receiving attention (62). In CF lung disease, although neutrophils can infiltrate and control chronic infections in the airways, there is evidence that NETs contribute to exacerbation of lung tissue damage, and the complete etiology is still not clear (63). In addition, with the outbreak of COVID-19 in 2020, research on NETs and severe acute respiratory syndrome coronavirus (SARS-CoV-2) has become the latest hot spot. Although NETs and NETosis can control the severity of viral infections (64), studies have shown that the persistence of NETs during respiratory viral infections can lead to tissue damage (65). In COVID-19, elevated levels of NETs may not only be associated with more severe ALI and ARDS (66–68) but may also lead to coagulation disorders (69). The mechanism of the increase in NET content in COVID-19 is still unclear; it may be related to SARS-CoV-2-mediated downregulation of cytokine storms (CSs) and angiotensin-converting enzyme 2 (ACE2), thereby inhibiting neutrophil infiltration (66, 70). In addition, there is evidence that ROS generated by SARS-CoV-2 infection can promote the generation of NETs (71).

Due to the negative impact of excessive NETs in many diseases, detection of NETs as biomarkers of disease and targeted reduction of NETs to treat related diseases have also become recent hotspots (13). In colorectal and breast cancer patients, measurement of NET-associated products including citH3 and MPO in the blood cfDNA is more specific than cfDNA alone (72, 73). Thalin et al. observed that high levels of citH3 in plasma are an important indicator of short-term mortality in some cancer patients (74). Further clinical studies are needed to clarify the link between the level of NETs and poor cancer/disease prognosis. Many studies have also tried different attempts to attenuate NETs formation for treating inflammatory diseases. Looney et al. proved that pretreatment with a non-steroidal anti-inflammatory drug aspirin could reduce endotoxin-induced acute lung injury by reducing the production of NETs (75). Lapponi et al. also demonstrated that aspirin could inhibit the inflammatory transcriptional regulator NF-κB that promotes NETosis (76). Diabetes is associated with an excess release of NETs and enhanced NETosis (77). Menegazzo et al. studied the effect of metformin, a drug commonly used to treat diabetes, on NETosis and found that compared with placebo, metformin significantly reduced NETs levels *in vitro (* 78). Further studies found that metformin prevented membrane translocation of PKC-βII and activation of NOX in neutrophils, thereby altering the pathological changes in nuclear dynamics and DNA release (78). Notably, Khan et al. screened 126 compounds, which belong to 39 classes commonly used for treating cancer, blood cell disorders, and other diseases, for NETosis modulating ability, and suggested anthracyclines along with dexamethasone as therapeutic candidates for suppressing unwanted NETosis in NETs overexpressing diseases (79). At present, the development of drugs to treat related diseases by targeting the formation or removal of NETs mainly focuses on basic research, and more clinical studies are needed to clarify the clinical effects of these drugs. In the future, developing specific and effective drugs to treat NETs-related diseases by targeting NETs formation is still a research hotspot in this field (13, 80, 81).

#### Neutrophil extracellular traps and cell death

As shown in **Figure 8**, the link between NETs and cell death is one of the emerging hotspots in recent years. To date, studies related to NETs and cell death have focused on the fields of NETosis, apoptosis, necroptosis, autophagy, and pyroptosis (82, 83). NETosis-related research started later and is considered a novel cell death mode differentiated from apoptosis and necrosis (82). Brinkmann Volker et al. are the first to discover that phorbol 12-myristate 13-acetate (PMA)-mediated release of NETs is accompanied by the neutrophil suicide, which was named “NETosis” (4). Studies have shown that a variety of molecular substances are involved in the regulation of NETosis (84). For example, NADPH oxidase, reactive oxygen species (ROS), was shown to be crucial in NETosis, promoting the formation and release of NETs (7, 10). Conversely, the exposure of neutrophils with the inhibitor of PAD4 citrullinating histones is associated with impaired NETosis and reduced release of NETs (11). It is worth mentioning that the release of NETs is not always accompanied by cell death, and living neutrophils can release components of NETs (14). In terms of apoptosis, evidence suggests that the formation of NETs does not occur in the process of apoptosis (8, 85), but some studies suggest that the disturbance of apoptosis and the removal of apoptotic cells may affect the release of NETs (86). Interestingly, Nades Palaniyar et al. discovered a novel form of cell death in which apoptosis and NETosis occur simultaneously in the same neutrophil (87). Under the condition of high doses of UV irradiation, they found that the activation of neutrophil apoptosis pathway was accompanied by the release of NETs, which they named “ApoNETosis” (88). In addition, if apoptotic neutrophils are not cleared by phagocytes in time, secondary necrotic/pyroptotic cell death may occur under the action of gasdermin family proteins (89). It has been suggested that this necrotic/pyroptotic may lead to the release of NETs (86), but further research is needed. There is no clear conclusion as to whether necroptosis is involved in the release of NETs. Mary Speir et al. found that receptor-interacting serine/threonine-protein kinase 1 (RIPK1) kinase-dependent necroptosis can promote plasma membrane degradation and DNA release by recruiting mixed lineage kinase domains such as pseudokinase (MLKL), which may favor the release of NETs (90). By contrast, Elaine F Kenny et al. induced neutrophil necroptosis by agonists such as TNF-α and Z-VAD-FMK and found no increase in the release of NETs (91). The relationship between NETs and autophagy is also controversial. On the one hand, in animal experiments, the lack of WDFY3 (WD Repeat and FYVE Domain Containing 3), a master regulator of selective autophagy, inhibits the generation of NETs (92). Eleni Frangou et al. found that patients with active SLE have enhanced levels of neutrophil autophagy, which leads to increased release of NETs (93). On the other hand, deletion of the autophagosome-forming gene autophagy-related 5 (ATG5) did not affect the ability of mouse neutrophils to form NETs (94). In addition, some signaling pathways play important roles in NETs and autophagy. For example, Asako Itakura et al. found that inhibition of the mammalian target of rapamycin (mTOR) enhanced autophagosome formation and accelerated the release of NETs, suggesting that the mTOR pathway may play a role in the release of NETs by regulating autophagic activity (95). Gasdermin D (GSDMD) plays an important role in pyroptosis (96). Many studies have shown that GSDMD can form functional pores within the cell membrane to disrupt the integrity of the membrane, leading to the occurrence of pyroptosis (97, 98). GSDMD is also crucial in NETs and pyroptosis (99). For example, Kaiwen W Chen et al. found that GSDMD-induced neutrophil death promotes the release of NETs under noncanonical (caspase-4/11) inflammasome signaling (100). Similarly, Gabriel Sollberger et al. demonstrated that the formation of NETs and the release of chromatin structures are dependent on GSDMD, which plays an important role in NETosis (101).

## Limitations

This study is the first to use bibliometric visualization to analyze NETs-related research in the past 20 years. However, this study inevitably has certain limitations. First, the data used in this study are only from the WOSCC database and do not include data from other databases, such as PubMed, Cochrane Library, and Google Scholar. Despite the comprehensiveness and reliability of the WOSCC, data from the WOSCC database may have certain missing articles. Second, only English-language literature was included in this study, which may lead to biased results. Finally, the data in this study may be inconsistent in various aspects; for example, the same institution may have used different names at different time periods.

## Conclusions

In this study, we used bibliometric analysis to review the trends, hot spots, and frontiers of NETs-related research in the last two decades. Frontiers In Immunology, Blood, etc., are influential journals in this field, and Brinkmann Volker is the authoritative author in this field. HEMATOLOGY, IMMUNOLOGY, and CELL DEATH are hot topics in this field, and the relationship between NETs and SARS-CoV-2 may be a direction for future research. Our study illustrates basic scientific knowledge and various interrelationships about extra neutrophil traps and provides important clues about research trends and frontiers. We hope this study will help researchers better grasp current general trends in the field.

## Data availability statement

The original contributions presented in this study are included in the article/**Supplementary Material**. Further inquiries can be directed to the corresponding authors.

## Author contributions

JL: study conception. YW, JS, and JO: study design. YW, JS, JO, YL, PD, YH and LL: study conduct. YW: data analysis. YW, JS, and JO: full access to all the data in the study, take responsibility for the integrity of the data and the accuracy of the data analysis, data interpretation, and drafting of the manuscript. YW, JS, JO, YL, PD, YH, LL, JL: critical revision of the manuscript for important intellectual content. All authors contributed to the article and approved the submitted version.

## Funding

This work was supported by the National Natural Science Foundation of China (82130063); Guangdong Basic and Applied Basic Research Foundation (No. 2020A1515011258, 2022A1515010529); Science and Technology Program of Guangzhou, China (No. 201904010382); and the College Students Innovation And Entrepreneurship Training Program Project, Southern Medical University (grant no. 202112121203).

## Conflict of interest

The authors declare that the research was conducted in the absence of any commercial or financial relationships that could be construed as a potential conflict of interest.

## Publisher’s note

All claims expressed in this article are solely those of the authors and do not necessarily represent those of their affiliated organizations, or those of the publisher, the editors and the reviewers. Any product that may be evaluated in this article, or claim that may be made by its manufacturer, is not guaranteed or endorsed by the publisher.
