# Corrigendum: Bibliometric and visual analysis of neutrophil extracellular traps from 2004 to 2022

**DOI:** 10.3389/fimmu.2022.1098082

**Published:** 2022-12-08

**Authors:** Yantong Wan, Junyi Shen, Jiafu Ouyang, Peng Dong, Yinghao Hong, Lixin Liang, Jinghua Liu

**Affiliations:** ^1^ Guangdong Provincial Key Laboratory of Proteomics, Department of Pathophysiology, School of Basic Medical Sciences, Southern Medical University, Guangzhou, China; ^2^ The Second Clinical Medical College, Southern Medical University, Guangzhou, China; ^3^ College of Anesthesiology, Southern Medical University, Guangzhou, China

**Keywords:** NETs, neutrophil, VOSviewer, CiteSpace, visual analysis

In the published article, there was an error in **Figure 3** as published. “Univ Erlangen Nurnberg”, “Friedrich Alexander Univ Erlan” and “Univ Klinikum Erlangen” were regarded as three independent institutions in our data analysis, but actually they are the same institution “Friedrich Alexander Univ Erlan”. The corrected **Figure 3** combines the data of “Univ Erlangen Nurnberg”, “Friedrich Alexander Univ Erlan” and “Univ Klinikum Erlangen” and its caption “Analysis of NETs-related institutions” appear below.

**Figure 3:**
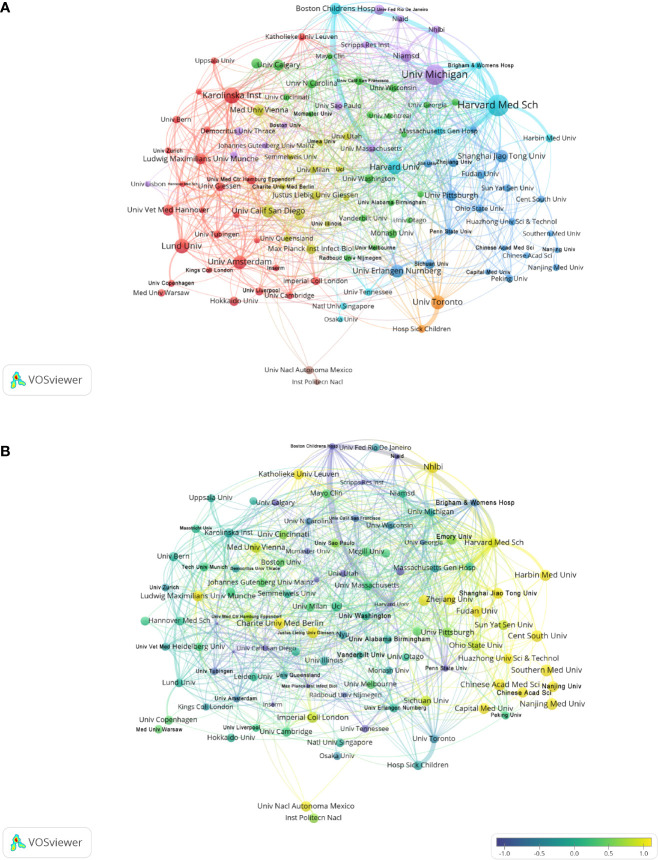
Analysis of NETs-related institution. **(A)** Analysis of collaborative network visualization of institutions in VOSviewer. The figure shows the institutions with more than 5 documents. The nodes of different colors represent the institutions of different clusters, and the size of the nodes indicates the frequency of their occurrence. **(B)** Analysis of the number of articles published by institutions in recent years. The recent 5 years heat value of each institution is obtained by dividing the number of publications in recent 5 year by the total number of publications.

In the published article, there was an error in

**Table 3** as published. **Table 3**, Rank 2, Martin Herrmann’s institution is University Hospital Würzburg (Germany). The correct institution should be University Hospital Erlangen (Germany). The corrected **Table 3** and its caption “Top 10 authors in terms of number of publications, the corresponding institutions and total link strength” appear below.

**Table 3:** Top 10 authors in terms of number of publications, the corresponding institutions and total link strength.

Rank	Author	Publications	Institutions	Total link Strength
1	Kaplan, Mariana J.	67	National Institutes of Health(USA)	247
2	Herrmann, Martin	51	University Hospital Erlangen(Germany)	361
3	Knight, Jason S.	49	University of Michigan(USA)	257
4	Von Koeckritz-Blickwede, Maren	47	University of Veterinary Medicine(Germany)	149
5	Nizet, Victor	41	University of Rhode Island College of Pharmacy(USA)	112
6	Wagner, Denisa D.	36	Boston Children’s Hospital(USA)	136
7	Hermosilla, Carlos	32	Justus Liebig University(Germany)	129
8	Taubert, Anja	32	Justus Liebig University(Germany)	134
9	Nakazawa, Daigo	31	Hokkaido University(Japan)	166
10	Ritis, Konstantinos	31	Democritus University of Thrace(Greece)	191

In the published article, there was an error. Professor Martin Herrmann’s institution is wrong.

A correction has been made to **Results**, *Distribution of authors*, Paragraph 1. This sentence previously stated:

“The author with the highest number of publications is Kaplan Mariana J. (National Institutes of Health, USA) (67), followed by Herrmann Martin (University Hospital Würzburg, Germany) (51), Knight Jason S. (University of Michigan, USA) (49) and Maren von Köckritz-Blickwede (University of Veterinary Medicine, Germany) (47).”

The corrected sentence appears below:

“The author with the highest number of publications is Kaplan Mariana J. (National Institutes of Health, USA) (67), followed by Herrmann Martin (University Hospital Erlangen, Germany) (51), Knight Jason S. (University of Michigan, USA) (49) and Maren von Köckritz-Blickwede (University of Veterinary Medicine, Germany) (47).”

The authors apologize for these errors and state that this does not change the scientific conclusions of the article in any way. The original article has been updated.

## Publisher’s note

All claims expressed in this article are solely those of the authors and do not necessarily represent those of their affiliated organizations, or those of the publisher, the editors and the reviewers. Any product that may be evaluated in this article, or claim that may be made by its manufacturer, is not guaranteed or endorsed by the publisher.

