# Supplementary material for: Bibliometric and visual analysis of neutrophil extracellular traps from 2004 to 2022
Source: Front Immunol. 2022 Oct 21;13:1025861. doi: 10.3389/fimmu.2022.1025861 (PMC9634160; doi:10.3389/fimmu.2022.1025861)
Supplement: Supplementary file 1 [file DataSheet_1.docx]

Supplementary Tables and Figures

## 1. Supplementary Tables

**Supplementary Table 1** | Top 10 institutions in terms of number of frequency of citations and the corresponding centrality.

| **Rank** | **Institution** | **Citations** | **Centrality** |
| --- | --- | --- | --- |
| 1 | Max Planck Institute for Infection Biology | 17982 | 0.09 |
| 2 | Harvard University | 12087 | 0.1 |
| 3 | University of Michigan | 10752 | 0.07 |
| 4 | University of Calgary | 8049 | 0.02 |
| 5 | New York University | 7783 | 0.02 |
| 6 | University of California, San Diego | 5266 | 0.06 |
| 7 | Boston Children's Hospital | 5247 | 0.05 |
| 8 | University of Munich | 5045 | 0.04 |
| 9 | Harvard Medical School | 4969 | 0.02 |
| 10 | Brigham and Women’s Hospital | 4660 | 0.21 |

**Supplementary Table 2** | Top 10 authors in terms of number of frequency of co-citations, the corresponding institutions and total link strength.

| **Rank** | **Author** | **Co-citations** | **Institutions** | **Total link Strength** |
| --- | --- | --- | --- | --- |
| 1 | Brinkmann, V | 4008 | Max Planck Institute(Germany) | 64468 |
| 2 | Fuchs, Ta | 2621 | New York University(USA) | 54054 |
| 3 | Papayannopoulos, V | 1690 | The Francis Crick Institute(UK) | 34418 |
| 4 | Urban, Cf | 1063 | Max Planck Institute(Germany) | 23086 |
| 5 | Hakkim, A | 898 | Harvard Medical School(USA) | 22119 |
| 6 | Clark, Sr | 889 | The University of Adelaide(Australia) | 19800 |
| 7 | Yipp, Bg | 764 | University of Calgary(Canada) | 20349 |
| 8 | Martinod, K | 729 | Harvard Medical School(USA) | 19306 |
| 9 | Wang, Ym | 678 | Capital Medical University(China) | 15872 |
| 10 | Yousefi, S | 673 | University of Bern(Switzerland) | 18906 |

**Supplementary Table 3** | Top 10 journals in terms of number of frequency of co-citations, corresponding IF (JCR2021) and JCR quartile.

| **Rank** | **Journal** | **Co-citations** | **IF(JCR2021)** | **JCR quartile** |
| --- | --- | --- | --- | --- |
| 1 | Blood | 14576 | 25.476 | Q1 |
| 2 | J Immunol | 13208 | 5.446 | Q2 |
| 3 | P Natl Acad Sci Usa | 8160 | 12.779 | Q1 |
| 4 | Plos One | 8085 | 3.752 | Q2 |
| 5 | Front Immunol | 7774 | 8.786 | Q1 |
| 6 | Science | 7237 | 63.798 | Q1 |
| 7 | Nat Med | 6956 | 87.241 | Q1 |
| 8 | J Clin Invest | 6320 | 19.486 | Q1 |
| 9 | J Exp Med | 5854 | 17.579 | Q1 |
| 10 | J Biol Chem | 5778 | 5.486 | Q2 |

**Supplementary Table 4** | Cooccurrence frequency, Burst, Degree, Centrality and Cluster_ID of the occurrence points in Figure 6B.

| **Cluster_ID** | **Keyword** | **Freq** | **Burst** | **Degree** | **Centrality** |
| --- | --- | --- | --- | --- | --- |
| 0 | in vivo | 182 | 19.95 | 20 | 0.03 |
| 0 | p selectin | 133 | 7.08 | 21 | 0.03 |
| 0 | blood | 59 | 5.32 | 23 | 0.03 |
| 0 | deep vein thrombosis | 171 | 3.88 | 23 | 0.04 |
| 0 | acute myocardial infarction | 17 | 4.31 | 9 | 0.04 |
| 0 | risk factor | 91 | 4.22 | 16 | 0.04 |
| 0 | ischemia reperfusion injury | 38 | 4.5 | 8 | 0.02 |
| 0 | biomarker | 28 | 4.57 | 6 | 0.01 |
| 0 | respiratory distress syndrome | 49 | 3.47 | 7 | 0.01 |
| 0 | disseminated intravascular coagulation | 43 | 5.49 | 8 | 0 |
| 0 | extracellular histone | 21 | 6.74 | 7 | 0 |
| 0 | antiphospholipid syndrome | 31 | 4.52 | 6 | 0 |
| 0 | cell-free dna | 26 | 3.74 | 7 | 0 |
| 0 | management | 23 | 5.19 | 6 | 0 |
| 0 | lipopolysaccharide | 17 | 4.31 | 10 | 0 |
| 0 | generation | 31 | 6.99 | 5 | 0 |
| 0 | risk | 58 | 4.25 | 4 | 0 |
| 0 | citrullinated histone h3 | 27 | 6.09 | 5 | 0 |
| 0 | therapy | 38 | 5.66 | 5 | 0 |
| 0 | severe sepsis | 20 | 7.12 | 5 | 0 |
| 0 | activated protein c | 11 | 5.01 | 7 | 0 |
| 0 | glycation end product | 5 |  | 1 | 0 |
| 0 | endothelial cell | 158 |  | 20 | 0.03 |
| 0 | tissue factor | 188 |  | 21 | 0.05 |
| 0 | serum | 7 |  | 1 | 0 |
| 0 | atherosclerosis | 94 |  | 13 | 0.02 |
| 0 | acute coronary syndrome | 8 |  | 2 | 0 |
| 0 | free plasma dna | 6 |  | 1 | 0 |
| 0 | septic shock | 70 |  | 16 | 0.03 |
| 0 | venous thrombosis | 91 |  | 16 | 0.04 |
| 0 | coagulation | 99 |  | 17 | 0.02 |
| 0 | trap | 46 |  | 6 | 0 |
| 0 | high glucose | 5 |  | 1 | 0 |
| 0 | thrombin generation | 38 |  | 9 | 0.01 |
| 0 | venous thromboembolism | 89 |  | 12 | 0.01 |
| 0 | acute lung injury | 192 |  | 25 | 0.05 |
| 0 | acute ischemic stroke | 8 |  | 1 | 0 |
| 0 | cell free fetal | 1 |  | 2 | 0 |
| 0 | amyloid p component | 1 |  | 2 | 0 |
| 0 | alpha | 6 |  | 7 | 0 |
| 0 | red blood cell | 6 |  | 1 | 0 |
| 0 | myocardial infarction | 64 |  | 12 | 0.02 |
| 0 | circulation | 1 |  | 2 | 0 |
| 0 | von willebrand factor | 98 |  | 16 | 0.02 |
| 0 | elevation | 1 |  | 2 | 0 |
| 0 | ischemic stroke | 30 |  | 6 | 0 |
| 0 | adhesion | 50 |  | 15 | 0.01 |
| 0 | blood platelet | 4 |  | 4 | 0 |
| 0 | coronary artery disease | 12 |  | 4 | 0 |
| 0 | platelet | 125 |  | 22 | 0.03 |
| 0 | extracellular vesicle | 14 |  | 4 | 0 |
| 0 | procoagulant activity | 10 |  | 3 | 0 |
| 0 | circulating dna | 15 |  | 6 | 0 |
| 0 | antigen g expression | 1 |  | 2 | 0 |
| 0 | tissue factor expression | 6 |  | 2 | 0.01 |
| 0 | actin | 1 |  | 0 | 0 |
| 0 | activated platelet | 82 |  | 14 | 0.02 |
| 0 | complement activation | 19 |  | 6 | 0 |
| 0 | c reactive protein | 60 |  | 12 | 0.03 |
| 0 | marker | 20 |  | 10 | 0.01 |
| 0 | sterile inflammation | 5 |  | 2 | 0 |
| 0 | arterial thrombosis | 11 |  | 4 | 0 |
| 0 | cardiovascular disease | 55 |  | 13 | 0.03 |
| 0 | microparticle | 13 |  | 5 | 0 |
| 0 | acute phase protein | 1 |  | 2 | 0 |
| 0 | beta(2) glycoprotein 1 | 1 |  | 2 | 0 |
| 0 | plasma | 41 |  | 20 | 0.07 |
| 0 | low density lipoprotein | 7 |  | 1 | 0 |
| 0 | ischemia/reperfusion injury | 7 |  | 1 | 0 |
| 0 | mortality | 32 |  | 7 | 0.01 |
| 0 | critically ill patient | 6 |  | 6 | 0.01 |
| 0 | antiphospholipid antibody | 17 |  | 6 | 0.02 |
| 1 | netting neutrophil | 137 | 12.45 | 21 | 0.03 |
| 1 | plasmacytoid dendritic cell | 70 | 5.05 | 19 | 0.03 |
| 1 | peptidylarginine deiminase inhibition | 82 | 5.23 | 17 | 0.03 |
| 1 | cathepsin g | 30 | 7.54 | 12 | 0.02 |
| 1 | antibody | 73 | 3.37 | 17 | 0.03 |
| 1 | disease activity | 66 | 5.69 | 13 | 0.02 |
| 1 | cytokine | 43 | 3.1 | 12 | 0.02 |
| 1 | polymorphonuclear leukocyte | 15 | 4.55 | 10 | 0.02 |
| 1 | autoantibody | 60 | 3.38 | 11 | 0.01 |
| 1 | i interferon | 17 | 3.64 | 7 | 0.01 |
| 1 | anca associated vasculiti | 12 | 4.27 | 8 | 0.01 |
| 1 | small vessel vasculiti | 17 | 6.05 | 8 | 0 |
| 1 | microscopic polyangiiti | 20 | 8.21 | 7 | 0 |
| 1 | autoimmune | 35 | 5.36 | 7 | 0 |
| 1 | autoimmunity | 27 | 9.61 | 3 | 0 |
| 1 | porphyromonas gingivali | 13 | 4.76 | 3 | 0 |
| 1 | damage | 69 |  | 12 | 0.01 |
| 1 | colony stimulating factor | 57 |  | 10 | 0.01 |
| 1 | smooth muscle cell | 6 |  | 1 | 0 |
| 1 | pathogenesis | 191 |  | 18 | 0.02 |
| 1 | b cell | 28 |  | 7 | 0 |
| 1 | wegeners granulomatosis | 5 |  | 2 | 0 |
| 1 | monocyte | 13 | 5.34 | 3 | 0 |
| 1 | interferon gamma | 4 |  | 3 | 0 |
| 1 | double blind | 9 |  | 2 | 0 |
| 1 | rheumatoid arthriti | 203 |  | 19 | 0.03 |
| 1 | low density granulocyte | 12 |  | 8 | 0.01 |
| 1 | peptidylarginine deiminase | 55 |  | 14 | 0.04 |
| 1 | immune complexe | 12 |  | 7 | 0 |
| 1 | t cell | 133 |  | 14 | 0.02 |
| 1 | synovial fluid | 6 |  | 2 | 0 |
| 1 | signaling pathway | 6 |  | 1 | 0 |
| 1 | central nervous system | 5 |  | 1 | 0 |
| 1 | crohns disease | 5 |  | 4 | 0 |
| 1 | natural killer cell | 13 |  | 4 | 0 |
| 1 | antineutrophil cytoplasmic antibody | 30 |  | 12 | 0.03 |
| 1 | glomerulonephriti | 6 |  | 3 | 0 |
| 1 | toll like receptor | 86 |  | 9 | 0.01 |
| 1 | protein arginine deiminase | 7 |  | 2 | 0 |
| 1 | antineutrophil cytoplasmic autoantibody | 6 |  | 3 | 0 |
| 1 | polymorphonuclear neutrophil | 7 |  | 1 | 0 |
| 1 | neutrophil | 112 |  | 18 | 0.02 |
| 1 | group box 1 | 7 |  | 2 | 0 |
| 1 | erythematosus | 7 |  | 3 | 0 |
| 1 | systemic lupus erythematosus | 298 |  | 24 | 0.06 |
| 1 | bone marrow | 49 |  | 10 | 0.01 |
| 1 | collagen induced arthriti | 5 |  | 4 | 0 |
| 1 | lupus nephriti | 5 |  | 2 | 0 |
| 1 | disease | 236 |  | 16 | 0.04 |
| 1 | regulatory t cell | 71 |  | 15 | 0.03 |
| 1 | monoclonal antibody | 14 |  | 3 | 0 |
| 1 | suppressor cell | 47 |  | 9 | 0.01 |
| 1 | peptide | 7 |  | 2 | 0 |
| 1 | association | 47 |  | 10 | 0.01 |
| 1 | mesenchymal stem cell | 12 |  | 3 | 0 |
| 1 | clearance | 14 | 3.15 | 2 | 0 |
| 2 | group a streptococcus | 69 | 21.35 | 19 | 0.04 |
| 2 | expression | 501 | 11.38 | 32 | 0.07 |
| 2 | escape | 61 | 18.51 | 19 | 0.03 |
| 2 | streptococcus pneumoniae | 86 | 15.54 | 24 | 0.04 |
| 2 | binding | 59 | 5.05 | 30 | 0.08 |
| 2 | protein | 176 | 9.48 | 18 | 0.02 |
| 2 | phagocytosis | 78 | 5.23 | 19 | 0.04 |
| 2 | resistance | 45 | 4.79 | 13 | 0.03 |
| 2 | streptococcus | 18 | 9.47 | 5 | 0.01 |
| 2 | infection | 262 | 3.58 | 16 | 0.02 |
| 2 | obstructive pulmonary disease | 25 | 6.67 | 8 | 0.01 |
| 2 | lung | 27 | 4.21 | 6 | 0.01 |
| 2 | human polymorphonuclear leukocyte | 8 | 4.2 | 4 | 0.01 |
| 2 | allow | 24 | 10.54 | 8 | 0 |
| 2 | virulence | 38 | 5.1 | 10 | 0 |
| 2 | differentiation | 28 | 6.32 | 4 | 0 |
| 2 | gene | 42 |  | 10 | 0.01 |
| 2 | drosophila melanogaster | 3 |  | 5 | 0 |
| 2 | identification | 85 |  | 13 | 0.01 |
| 2 | capacitation | 1 |  | 1 | 0 |
| 2 | candida albican | 60 |  | 15 | 0.02 |
| 2 | receptor | 191 |  | 27 | 0.06 |
| 2 | cystic fibrosis | 66 |  | 15 | 0.02 |
| 2 | antimicrobial protein | 1 |  | 1 | 0 |
| 2 | escherichia coli | 32 |  | 23 | 0.04 |
| 2 | staphylococcus aureus | 143 |  | 20 | 0.04 |
| 2 | adhesion molecule | 1 |  | 1 | 0 |
| 2 | system | 5 |  | 1 | 0 |
| 2 | accumulation | 1 |  | 1 | 0 |
| 2 | recognition | 6 |  | 1 | 0 |
| 2 | neutrophil extracellular trap | 2700 |  | 35 | 0.08 |
| 2 | dnase | 7 |  | 8 | 0.02 |
| 2 | granule | 2 |  | 1 | 0 |
| 2 | active site | 1 |  | 1 | 0 |
| 2 | pimephales promelas rafinesque | 2 |  | 6 | 0 |
| 2 | diversity | 1 |  | 2 | 0 |
| 2 | metabolism | 6 |  | 1 | 0 |
| 2 | bactericidal activity | 7 |  | 6 | 0 |
| 2 | beta glucan | 2 |  | 6 | 0 |
| 2 | leukocyte | 4 |  | 9 | 0.01 |
| 2 | complete genome sequence | 1 |  | 2 | 0 |
| 2 | activation | 644 |  | 34 | 0.06 |
| 2 | extracellular dna | 12 |  | 6 | 0 |
| 2 | antimicrobial peptide ll 37 | 5 |  | 1 | 0 |
| 2 | innate immune response | 23 | 6.31 | 3 | 0 |
| 2 | apolipophorin iii | 1 |  | 1 | 0 |
| 2 | bombyx mori | 1 |  | 4 | 0 |
| 2 | epidemiology | 10 |  | 6 | 0 |
| 3 | chronic granulomatous disease | 40 | 14.13 | 13 | 0.02 |
| 3 | death | 103 | 4.91 | 17 | 0.02 |
| 3 | mouse | 27 | 6.09 | 9 | 0.02 |
| 3 | mitochondrial dna | 169 | 3.82 | 22 | 0.03 |
| 3 | tumor necrosis factor | 46 | 4.32 | 10 | 0.02 |
| 3 | degradation | 68 | 5.25 | 9 | 0.02 |
| 3 | histone | 103 | 3.52 | 17 | 0.02 |
| 3 | cell death | 196 | 4.66 | 17 | 0.01 |
| 3 | pad4 | 39 | 5.33 | 10 | 0.01 |
| 3 | extracellular trap formation | 87 | 3.79 | 11 | 0.01 |
| 3 | net formation | 90 | 3.41 | 13 | 0.01 |
| 3 | chromatin decondensation | 45 | 3.55 | 10 | 0.01 |
| 3 | citrullination | 20 | 7.13 | 5 | 0 |
| 3 | inhibitor | 43 | 4.15 | 9 | 0.01 |
| 3 | cell free dna | 36 | 4.63 | 7 | 0 |
| 3 | exposure | 15 | 6.16 | 4 | 0 |
| 3 | acid | 5 | 3.11 | 5 | 0 |
| 3 | chromatin | 17 | 8.77 | 5 | 0 |
| 3 | myeloperoxidase | 255 |  | 21 | 0.02 |
| 3 | dna trap | 129 |  | 18 | 0.02 |
| 3 | netosis | 299 |  | 19 | 0.02 |
| 3 | apoptosis | 173 |  | 30 | 0.05 |
| 3 | hypochlorous acid | 4 |  | 2 | 0 |
| 3 | gasdermin d | 15 | 4.15 | 5 | 0 |
| 3 | involvement | 6 |  | 1 | 0 |
| 3 | anca | 5 |  | 1 | 0 |
| 3 | necrosis factor alpha | 56 |  | 6 | 0 |
| 3 | hydrogen peroxide | 4 |  | 1 | 0 |
| 3 | net | 88 |  | 11 | 0.01 |
| 3 | mammalian target | 5 |  | 3 | 0 |
| 3 | innate | 85 |  | 12 | 0.01 |
| 3 | fibrosis | 8 |  | 1 | 0 |
| 3 | nadph oxidase | 239 |  | 23 | 0.04 |
| 3 | mechanism | 394 |  | 21 | 0.02 |
| 3 | immunity | 242 |  | 19 | 0.01 |
| 3 | inhibition | 141 |  | 22 | 0.05 |
| 3 | dna | 245 |  | 18 | 0.02 |
| 3 | elastase | 177 |  | 13 | 0.01 |
| 3 | induce | 9 | 4.18 | 4 | 0 |
| 3 | role | 8 |  | 1 | 0 |
| 3 | nf kappa b | 109 |  | 17 | 0.03 |
| 3 | tnf alpha | 26 |  | 6 | 0 |
| 3 | susceptibility | 6 |  | 1 | 0 |
| 3 | aspirin | 4 |  | 3 | 0 |
| 3 | antimicrobial activity | 8 | 5.16 | 3 | 0 |
| 3 | release | 348 |  | 27 | 0.05 |
| 3 | reactive oxygen | 4 |  | 3 | 0 |
| 3 | nlrp3 inflammasome | 87 |  | 10 | 0.01 |
| 4 | innate immunity | 450 | 18 | 28 | 0.05 |
| 4 | antimicrobial peptide | 54 | 8.99 | 19 | 0.04 |
| 4 | host defense | 134 | 7.44 | 23 | 0.03 |
| 4 | in vitro | 177 | 3.54 | 21 | 0.06 |
| 4 | apoptotic cell | 35 | 3.96 | 21 | 0.04 |
| 4 | epithelial cell | 38 | 3.85 | 18 | 0.04 |
| 4 | macrophage | 156 | 3.7 | 21 | 0.03 |
| 4 | autoimmune disease | 43 | 5.17 | 16 | 0.02 |
| 4 | gene expression | 74 | 5.34 | 11 | 0.01 |
| 4 | granulocyte | 25 | 3.19 | 15 | 0.02 |
| 4 | adaptive immunity | 18 | 4.83 | 9 | 0 |
| 4 | mycobacterium tuberculosis | 12 | 4.92 | 5 | 0 |
| 4 | inflammatory bowel disease | 29 | 3.49 | 5 | 0 |
| 4 | intercellular adhesion molecule 1 | 6 |  | 2 | 0 |
| 4 | endothelial growth factor | 17 | 6.06 | 2 | 0 |
| 4 | macrophage polarization | 7 |  | 5 | 0.01 |
| 4 | virulence factor | 11 |  | 4 | 0 |
| 4 | arthriti | 6 |  | 1 | 0 |
| 4 | matrix metalloproteinase | 16 | 3.61 | 3 | 0 |
| 4 | dendritic cell | 191 |  | 22 | 0.06 |
| 4 | immune response | 126 |  | 12 | 0.01 |
| 4 | immune system | 22 | 4.52 | 4 | 0 |
| 4 | catfish ictalurus punctatus | 2 |  | 1 | 0 |
| 4 | aspergillus fumigatus | 2 |  | 3 | 0 |
| 4 | inflammatory response | 64 |  | 10 | 0.01 |
| 4 | reactive oxygen specy | 97 |  | 8 | 0.01 |
| 4 | mast cell | 23 |  | 6 | 0 |
| 4 | cytokine production | 20 |  | 7 | 0.01 |
| 4 | antibody therapeutics | 1 |  | 4 | 0 |
| 4 | lipopolysaccharide binding protein | 2 |  | 5 | 0 |
| 4 | molecular mechanism | 42 |  | 9 | 0 |
| 4 | inflammasome | 6 |  | 4 | 0 |
| 4 | alveolar macrophage | 4 |  | 6 | 0 |
| 4 | antimicrobial peptide protect | 1 |  | 2 | 0 |
| 4 | toll like receptor 4 | 3 |  | 4 | 0 |
| 4 | cerebral malaria | 2 |  | 4 | 0 |
| 4 | histone deimination | 9 |  | 2 | 0 |
| 4 | oxidative stress | 152 |  | 16 | 0.02 |
| 4 | ulcerative coliti | 15 |  | 4 | 0 |
| 4 | prevalence | 8 |  | 2 | 0 |
| 4 | c terminal domain | 1 |  | 4 | 0 |
| 4 | autophagy | 50 |  | 8 | 0.01 |
| 4 | pathogen | 2 |  | 2 | 0 |
| 4 | pattern recognition receptor | 13 |  | 2 | 0 |
| 4 | acute infection | 1 |  | 1 | 0 |
| 5 | cancer | 63 | 5.64 | 14 | 0.03 |
| 5 | circulating tumor cell | 45 | 4.94 | 14 | 0.03 |
| 5 | progression | 40 | 6.21 | 11 | 0.01 |
| 5 | respiratory burst | 37 | 3.68 | 11 | 0.02 |
| 5 | promote | 64 | 3.83 | 11 | 0.01 |
| 5 | interleukin 8 | 17 | 3.98 | 9 | 0.01 |
| 5 | pseudomonas aeruginosa | 25 | 5.41 | 7 | 0 |
| 5 | children | 18 | 3.38 | 6 | 0.01 |
| 5 | deficiency | 24 | 5.41 | 5 | 0 |
| 5 | growth | 23 | 5.19 | 7 | 0 |
| 5 | breast cancer | 32 | 4.66 | 3 | 0 |
| 5 | liver | 16 | 5.7 | 3 | 0 |
| 5 | immune cell | 20 | 7.13 | 3 | 0 |
| 5 | peptidylarginine deiminase 4 | 15 | 5.34 | 4 | 0 |
| 5 | chemotaxi | 12 |  | 7 | 0 |
| 5 | protect | 6 |  | 2 | 0 |
| 5 | proteinase 3 | 7 |  | 1 | 0 |
| 5 | metastasis | 19 |  | 5 | 0 |
| 5 | kill bacteria | 6 |  | 3 | 0 |
| 5 | migration | 50 |  | 10 | 0.01 |
| 5 | neutrophil elastase | 34 |  | 15 | 0.03 |
| 5 | oxidative burst | 15 | 5.34 | 2 | 0 |
| 5 | airway inflammation | 7 |  | 3 | 0 |
| 5 | serine protease | 14 |  | 4 | 0.01 |
| 5 | diagnosis | 7 |  | 2 | 0 |
| 5 | neutrophil activation | 6 |  | 2 | 0 |
| 5 | promote thrombin generation | 5 |  | 3 | 0 |
| 5 | to lymphocyte ratio | 7 |  | 3 | 0 |
| 5 | hepatocellular carcinoma | 7 |  | 3 | 0 |
| 5 | pathway | 68 |  | 10 | 0.02 |
| 5 | tumor microenvironment | 17 |  | 4 | 0 |
| 5 | mutation | 7 |  | 5 | 0.01 |
| 5 | neutrophil extracellular traps (nets) | 136 |  | 18 | 0.04 |
| 5 | acute kidney injury | 12 | 3.32 | 3 | 0 |
| 5 | tumor cell | 9 |  | 3 | 0 |
| 5 | high density lipoprotein | 6 |  | 2 | 0 |
| 5 | reveal | 7 |  | 3 | 0 |
| 5 | survival | 45 |  | 11 | 0.02 |
| 5 | quantification | 7 |  | 3 | 0 |
| 6 | mice | 173 | 9.57 | 22 | 0.05 |
| 6 | bacteria | 82 | 9.4 | 15 | 0.02 |
| 6 | extracellular trap | 435 | 3.45 | 20 | 0.02 |
| 6 | contribute | 71 | 5.05 | 10 | 0.01 |
| 6 | induction | 57 | 5.45 | 9 | 0.01 |
| 6 | classification | 18 | 4.05 | 7 | 0 |
| 6 | interferon | 9 |  | 1 | 0 |
| 6 | circulating histone | 8 |  | 3 | 0 |
| 6 | cell | 391 |  | 19 | 0.03 |
| 6 | mediator | 52 |  | 11 | 0.01 |
| 6 | sepsis | 105 |  | 20 | 0.03 |
| 6 | activated human platelet | 2 |  | 6 | 0 |
| 6 | resolution | 9 |  | 1 | 0 |
| 6 | acute respiratory distress syndrome | 7 |  | 2 | 0 |
| 6 | animal model | 8 |  | 2 | 0 |
| 6 | hmgb1 | 7 |  | 2 | 0 |
| 6 | anucleate platelet | 2 |  | 8 | 0 |
| 6 | thrombosis | 146 |  | 16 | 0.02 |
| 6 | molecular weight heparin | 8 |  | 2 | 0 |
| 6 | model | 55 |  | 9 | 0.01 |
| 6 | extracellular dna trap | 4 |  | 2 | 0 |
| 6 | polarization | 7 |  | 2 | 0 |
| 6 | recruitment | 119 |  | 16 | 0.04 |
| 6 | defense | 3 |  | 1 | 0 |
| 6 | dysfunction | 47 |  | 7 | 0 |
| 6 | inflammation | 507 |  | 30 | 0.05 |
| 6 | injury | 105 |  | 12 | 0.03 |
| 6 | manifestation | 5 |  | 3 | 0 |
| 6 | response | 63 |  | 12 | 0.02 |
| 6 | complement | 12 |  | 2 | 0 |
| 7 | sars coronavirus | 20 | 7.13 | 8 | 0.02 |
| 7 | endothelial dysfunction | 29 | 8.03 | 6 | 0.01 |
| 7 | cytokine storm | 33 | 9.14 | 5 | 0.01 |
| 7 | pneumonia | 17 | 6.06 | 5 | 0.01 |
| 7 | coronavirus | 27 | 7.47 | 5 | 0 |
| 7 | ace2 | 16 | 5.7 | 5 | 0 |
| 7 | platelet activation | 35 | 4.83 | 4 | 0 |
| 7 | nitric oxide | 32 | 3.08 | 8 | 0.01 |
| 7 | lymphocyte ratio | 8 |  | 2 | 0 |
| 7 | murine model | 8 |  | 1 | 0 |
| 7 | health | 7 |  | 2 | 0 |
| 7 | mouse model | 50 |  | 9 | 0.02 |
| 7 | covid 19 | 24 |  | 3 | 0 |
| 7 | blood brain barrier | 7 |  | 3 | 0 |
| 7 | coronavirus disease 2019 | 7 |  | 2 | 0 |
| 7 | insulin resistance | 7 |  | 4 | 0 |
| 7 | modulation | 7 |  | 4 | 0 |
| 7 | lung injury | 43 |  | 17 | 0.03 |
| 7 | acute respiratory syndrome | 8 |  | 2 | 0 |
| 7 | nitric oxide synthase | 11 |  | 6 | 0.01 |
| 7 | respiratory syndrome coronavirus | 7 |  | 2 | 0 |

## 2. Supplementary Figures


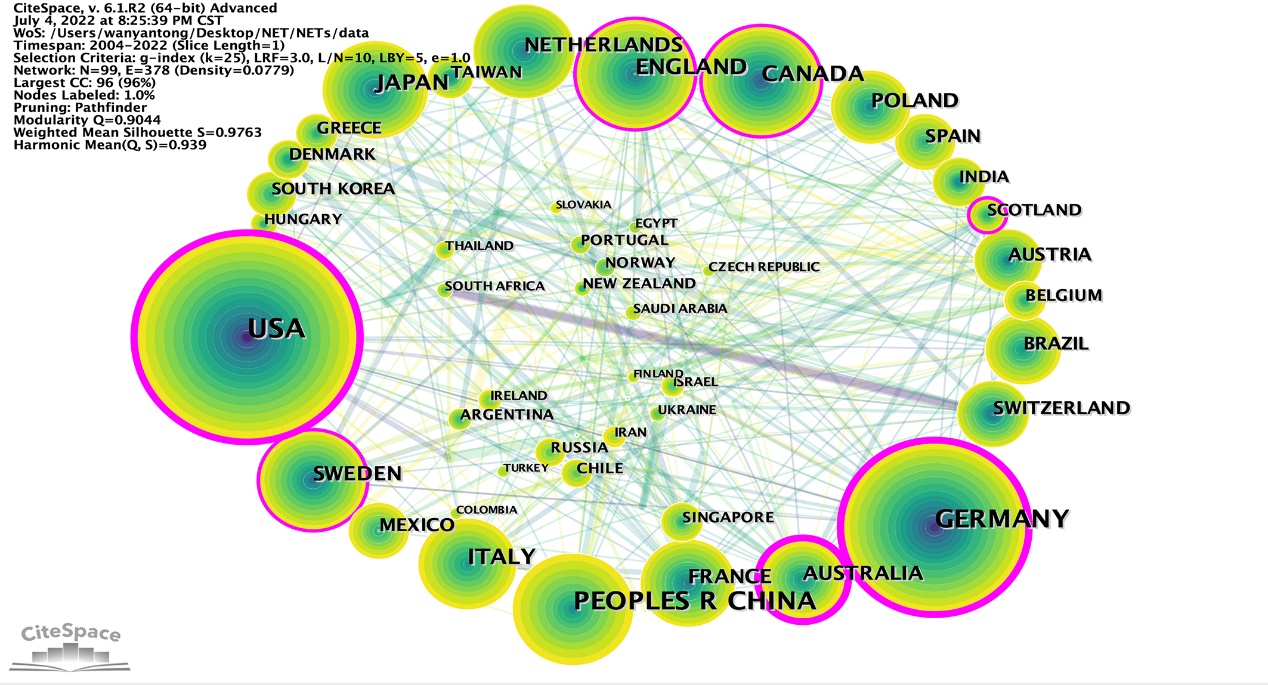


**Supplementary Figure 1** | Analysis of the collaboration network of countries/regions in CiteSpace.

**Supplementary Figure 1** shows collaboration network of countries/regions. The size of the nodes indicates the co-occurrence frequencies, and the links indicate the co-occurrence relationship. The nodes with purple outer circles represent their higher centrality.


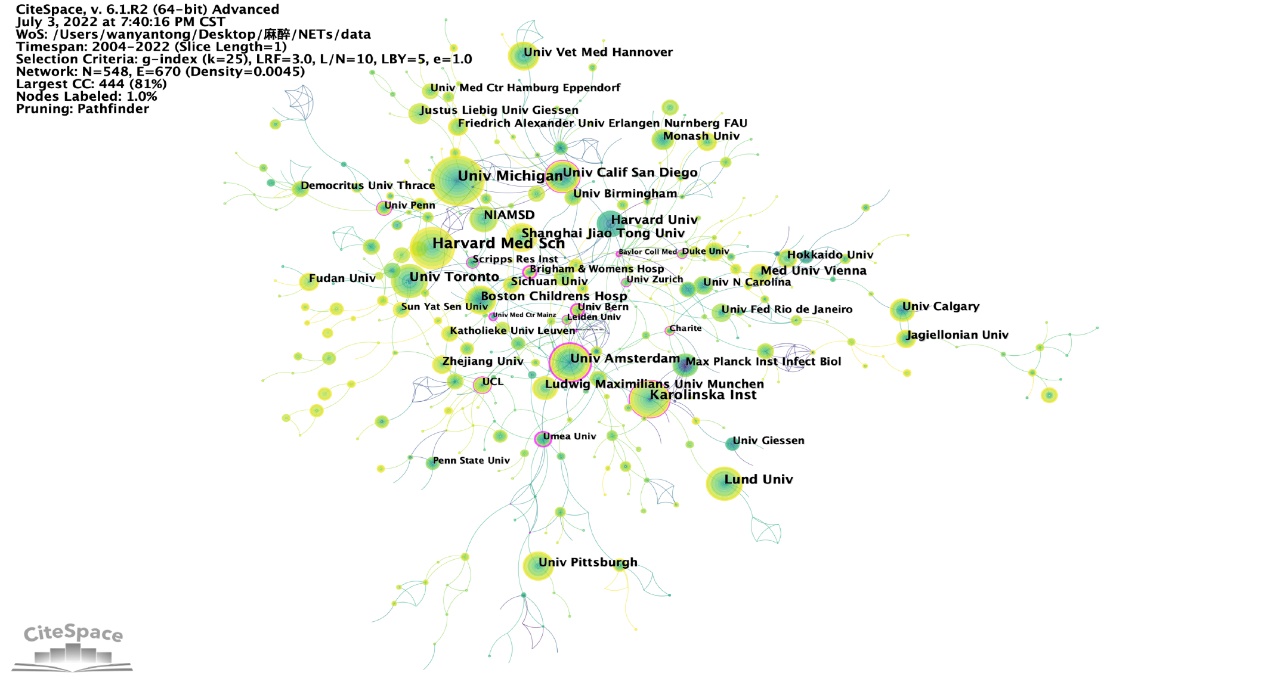


**Supplementary Figure 2** | Analysis of collaborative network visualization of institutions in CiteSpace.

**Supplementary Figure 2** shows collaborative network visualization of institutions. The size of the nodes indicates the co-occurrence frequencies, the links indicate the co-occurrence relationship, and the nodes with purple outer circles represent their higher centrality.


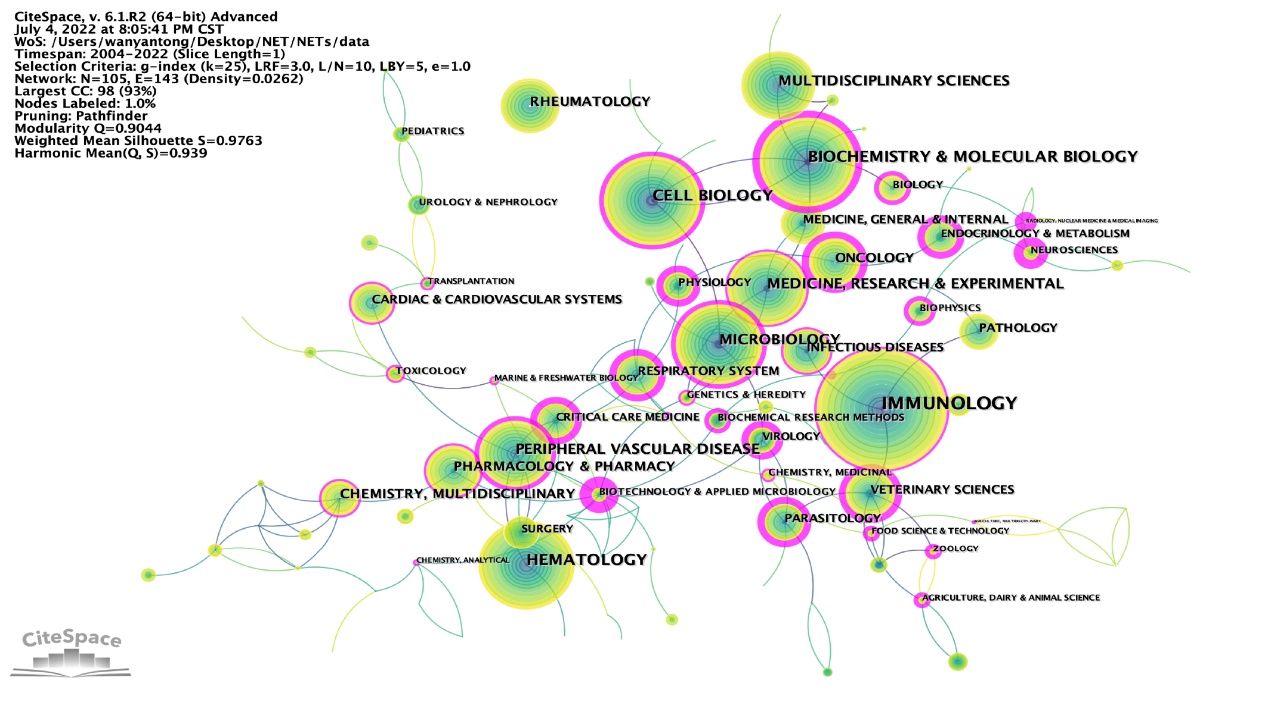


**Supplementary Figure 3** | Analysis of NETs correlation subject.

**Supplementary Figure 3** shows the analysis of NETs correlation subject. The size of the nodes indicates the co-occurrence frequencies, the links indicate the co-occurrence relationship, and the nodes with purple outer circles represent their higher centrality.


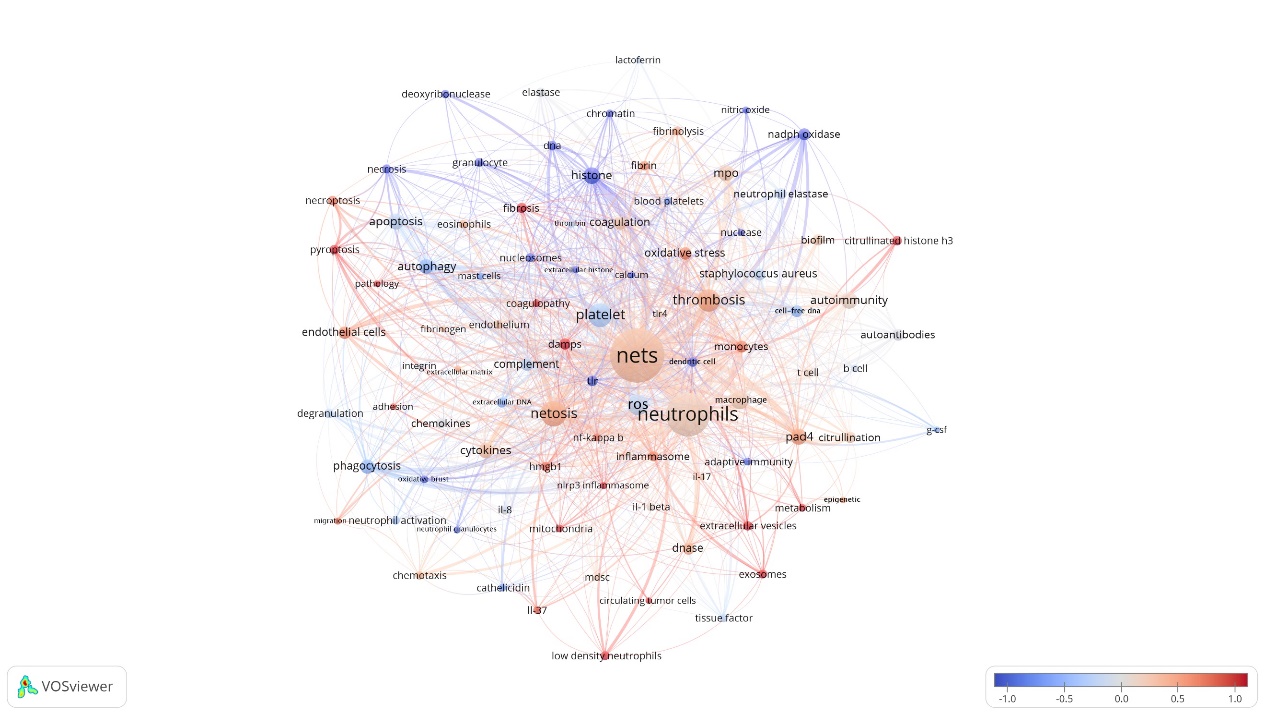


**Supplementary Figure 4** | Analysis of NETs correlation mechanism-related keywords.

**Supplementary Figure 4** shows the analysis of NETs mechanism-related keywords. The heat value of each mechanism-related keyword is the mean of the corresponding publication year. The color bias towards red means a higher value, indicating that these keywords are emerging forces in the field of NETs; the color bias towards blue means a lower value, indicating that these keywords have relatively a little research in the field of NETs in recent years. The results show that keywords such as hmgb1, exosomes, damps, extracellular vesicles have the later mean of publication year, indicating that they are newly discovered mechanisms in recent years. In contrast, the mean of the publication year for keywords such as TLR, nucleosomes, histone, and DNA is earlier, indicating that they are mechanisms that have long been discovered.


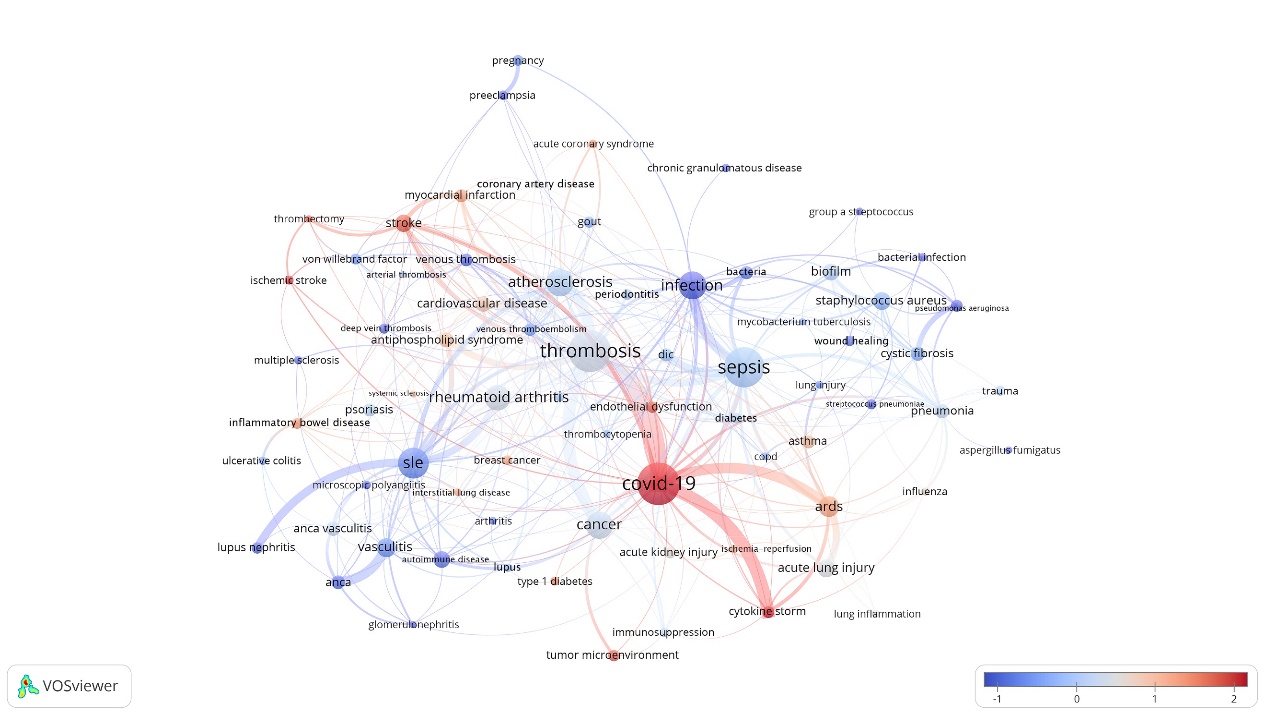


**Supplementary Figure 5** | Analysis of NETs correlation disease-related keywords.

**Supplementary Figure 5** shows the analysis of NETs disease-related keywords. The heat value of each disease-related keyword is the mean of the corresponding publication year. The color bias towards red means a higher value, indicating that these keywords are emerging forces in the field of NETs; the color bias towards blue means a lower value, indicating that these keywords have relatively a little research in the field of NETs in recent years. The results show that keywords such as COVID-19, cytokine storm have the later mean of publication year, indicating that they are newly discovered diseases in recent years. In contrast, the mean of the publication year for keywords such as infection, autoimmune disease, and vasculitis is earlier, indicating that they are disease that have long been discovered.
